# A parametric study of bubble dynamics and lesion formation in liver tissue phantom during pressure-modulated shockwave histotripsy

**DOI:** 10.1038/s41598-025-11512-x

**Published:** 2025-07-21

**Authors:** Jun Hong Park, Jeongmin Heo, Kisoo Pahk, Ki Joo Pahk

**Affiliations:** 1 Department of Radiology, Stanford University School of Medicine, Palo Alto, CA 94304 USA; 2 Bionics Research Center, Biomedical Research Institute, Korea Institute of Science and Technology (KIST), Seoul, 02792 Republic of Korea; 3 Department of Nuclear Medicine, Korea University College of Medicine, 73, Inchon-ro, Seongbuk-gu, Seoul, 02841 Republic of Korea; 4 Department of Biomedical Engineering, Kyung Hee University, Yongin, 17104 Republic of Korea

**Keywords:** HIFU, Boiling histotripsy, Pressure-modulated shockwave histotripsy, Bubble dynamics, Biomedical engineering, Therapeutics

## Abstract

Pressure-modulated shockwave histotripsy (PSH) is a new type of histotripsy that has recently been proposed for precise mechanical tissue fractionation without inducing the shock scattering effect. Though a previous initial proof-of-concept study clearly demonstrated the feasibility of PSH through introducing the pressure modulation time point (*t*_m_) to minimise the shock scattering effect, this study was limited to the examination of the effect of a single PSH pulse at a constant *t*_m_ on bubble dynamics. Here, we investigated the effects of varying PSH exposure conditions, particularly changes of *t*_m_ and the number of pulses, on bubble dynamics and lesion formation in liver tissue phantoms. Our experimental results reveal that a gradual increase in *t*_m_ beyond the time-to-boil can start to initiate shock scattering effect, thereby forming a larger PSH lesion with the transformation of the lesion shape of an ellipsoid to an elongated tadpole. Furthermore, the PSH lesion size gradually increased with PSH pulses. Under the 2 MHz PSH exposure, the smallest oval-shaped lesion area was measured to be 0.18 mm^2^ with *t*_m_ of 4 ms and a single PSH pulse, whereas the largest tadpole-shaped lesion area of 5.39 mm^2^ was produced with *t*_m_ of 9 ms and 50 PSH pulses. Taken together, these suggest that PSH could be a promising approach for effectively controlling bubble dynamics and lesion formation.

**Supplementary Information:**

The online version contains supplementary material available at 10.1038/s41598-025-11512-x.

## Introduction

Boiling histotripsy (BH) is a High-Intensity Focused Ultrasound (HIFU) technique which has shown potential for mechanical tissue fractionation without inducing thermal damage at the HIFU focus. BH has been investigated as a promising tool for treating cancer^1–3^ and other clinical applications including treatments of blood clots^4^ and liver cirrhosis^5^. The mechanisms of BH mainly involve shockwave heating followed by shock scattering effect. The use of milliseconds-long HIFU pulses with shockwaves at the HIFU focus used in BH can lead to rapid heating to boiling temperature within a few milliseconds (shockwave heating)^3,6–13^. A millimeter-sized boiling vapour bubble can then form, fractionating the surrounding tissue^6^. Furthermore, the boiling bubble inverts incoming shockwaves leading to the formation of additional bubble nucleation (shock scattering effect), generating an inertial cavitation cluster that progresses towards the HIFU transducer^14,15^. Multiple boiling bubbles formed within the localised shockwave heated region and the cavitation clusters result in the production of a tadpole-shaped BH lesion. The tail part of a BH lesion is generated by the shockwave heating-induced boiling bubble and the head, which is located toward the HIFU transducer, is due to the shock scattering-induced cavitation clouds^15^. The extent of mechanical damage generated by a boiling bubble and its further interaction with incoming incident shockwaves becomes larger than that of potential thermal injury by shockwave heating^11,13^. Hence, no thermal damage occurs after BH exposure. BH typically employs center frequencies in the range of 1 to 3 MHz with peak positive pressure (*P*_+_) of 67 to 85.4 MPa and peak negative pressure (*P*_–_) of 9 to 15.6 MPa^6,13^. Precise control of the extent and degree of mechanical damage induced by BH is necessary, especially when treating a solid tumour adjacent to major blood vessels. This is, however, difficult to achieve because of the generation of the shock scattering induced violent cavitation clouds during BH exposure.

In addition to the BH technique, pressure-modulated shockwave histotripsy (PSH) has recently been proposed for precise mechanical tissue fractionation^16, ^particularly for treating a solid tumor adjacent to normal tissue or major blood vessel (i.e., precise lesion control is essential for preserving surrounding vasculature), and its efficacy has been demonstrated in rat liver model in vivo^17^. It has been suggested that PSH could potentially be used to generate a spatially localised tissue destruction through controlling the size and lifetime of a boiling bubble without the shock scattering effect^16^. Shear stresses produced around this boiling bubble can mechanically fractionate surrounding tissue. Briefly, within a single PSH pulse, HIFU waves with high peak positive (*P*_1,+_) and negative pressure (*P*_1,–_) amplitudes at focus (i.e., shockwaves), which are comparable to those used in BH, are initially applied in order to produce a number of boiling vapour bubbles via shockwave heating. Subsequently, HIFU waves with relatively lower peak pressure amplitudes (*P*_2,+_ and *P*_2,–_) are employed to keep the boiling bubbles whilst avoiding or minimising the shock scattering effect^14,16^. *P*_2,+_ and *P*_2,–_ can limit the peak negative pressure of the reflected acoustic fields and thereby suppressing the formation of inertial cavitation. The pressure modulation time point (*t*_m_) used in PSH is the time point when the peak positive and negative pressures change from *P*_1,+_ and *P*_1,–_ to *P*_2,+_ and *P*_2,–_, and can be numerically or experimentally determined based upon the time to reach boiling temperature or form a boiling vapour bubble (i.e., time-to-boil) at a given *P*_1,+_ and *P*_1,–_, which is dependent upon thermal and acoustic properties of the target tissue^16^. The pressure modulation phase *P*_2,+_ and *P*_2,–_ within a single PSH pulse provides an effective means to control the extent and degree of boiling bubble-induced mechanical damage^16,17^. It has been shown that the maximum boiling bubble size as well as the lesion size measured at the end of the PSH pulse (at a given *P*_1,+_, *P*_1,–_, *t*_m_ and pulse length) were proportional to the magnitudes of *P*_2,+_ and *P*_2,–_, and the boiling bubble was persisted and maintained within the focal region until the end of the exposure^16^. Furthermore, a gradual increase in PSH pulse length (at a given *P*_1,+_, *P*_1,–_, *P*_2,+_, *P*_2,–_ and *t*_m_) can also gradually increase the degree of mechanical damage of liver tissue in rat’s liver in vivo (i.e., from tissue decellularisation to complete tissue fractionation with increasing PSH pulse length)^17^.

The use of multiple HIFU pulses is a common approach in histotripsy, which is one of the key parameters for varying the degree of mechanical damage produced^13,18,19^. Whilst the previous PSH proof-of-concept study^16^ has shown the effect of a pressure modulated HIFU pulse on bubble dynamics, this study was, however, limited to a single PSH pulse at a constant *t*_m_. An increase in *t*_m_ would essentially affect bubble dynamics via the shock scattering effect during PSH. To further demonstrate the potential effectiveness of PSH approach, the main objective of the present study is therefore to investigate and quantify the changes in bubble dynamics and lesion formation with increasing *t*_m_ (4 to 9 ms) and pulse number (1 to 50 pulses) during PSH exposure in an optically transparent liver tissue phantom. Quantitative analyses comparing the bubble size and corresponding lesion size as a function of *t*_m_ and pulse number are carried out.

A histotripsy clinical device (Edison^®^, HistoSonics, USA) to treat patients with liver tumours has recently been approved by the US Food and Drug Administration (FDA) in Oct 2023. Therefore, our results obtained with the liver tissue phantom are highly relevant to the treatment of liver diseases with pressure-modulated shockwave histotripsy technique.

## Results

### PSH using a 2 MHz HIFU transducer

**Fig. 1:**
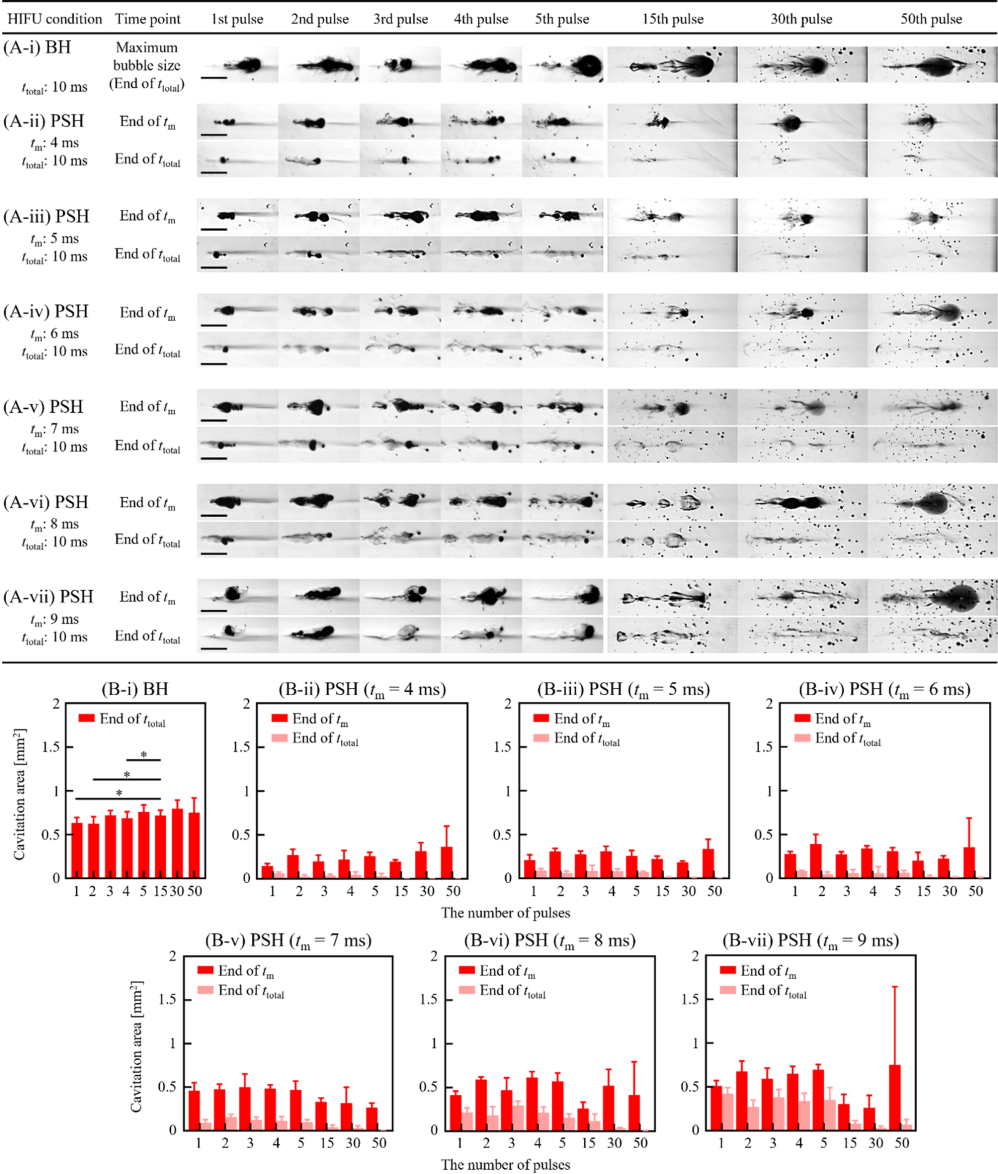
Cavitation dynamics induced by the 2 MHz BH and PSH exposure conditions at 1, 2, 3, 4, 5, 15, 30 and 50 pulses. High-speed camera images of bubble dynamics for **(A-i)** BH (*P*_+_ of 89.1 MPa, *P*_–_ of – 14.6 MPa) and **(A-ii to -vii)** for PSH (*P*_1,+_ of 89.1 MPa, *P*_1,–_ of – 14.6 MPa, *P*_2,+_ of 29.9 MPa and *P*_2,–_ of – 9.6 MPa) at the end of *t*_m_ and *t*_total_ at each pulse. In PSH, *t*_m_ varied from 4 to 9 ms within *t*_total_ of 10 ms. A DC of 1% and a pulse repetition frequency of 1 Hz were used. A scale bar indicates 1 mm. The HIFU beam propagates from left to right. Boiling bubbles were produced within the localised shockwaves heated region. A movie showing the cavitation dynamics captured during the 2 MHz BH and PSH exposures is available in Supplementary Video S1. **(B)** Cavitation bubble area measured at *t*_m_ and *t*_total_ with *t*_m_ of 4 to 9 ms and the number of pulses from 1 to 50 pulses (*p < 0.05).

**Fig. 2:**
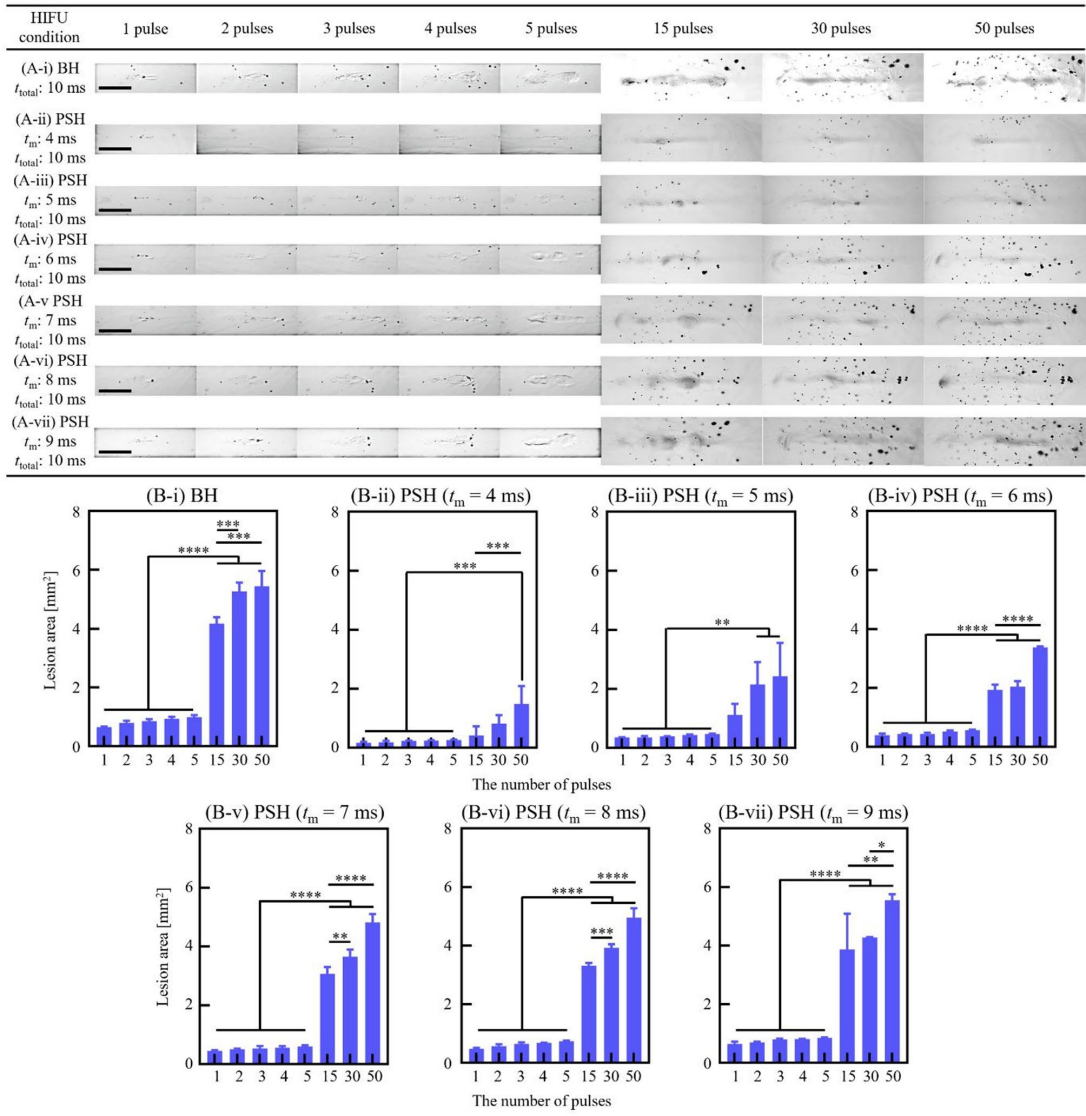
BH and PSH lesion areas produced by the 2 MHz HIFU transducer with varying *t*_m_ and number of pulses. High-speed camera images of the lesion formation in the liver tissue phantom under various exposure conditions: **(A-i)** BH exposure conditions with a *t*_total_ of 10 ms. PSH exposure conditions with *t*_m_ of **(A-ii)** 4 ms, **(A-iii)** 5 ms, **(A-vi)** 6 ms, **(A-v)** 7 ms, **(A-vi)** 8 ms and **(A-vii)** 9 ms with the number of pulses from 1, 2, 3, 4, 5, 15, 30 and 50 pulses. Images (A-i to A-vii) were taken after the corresponding PSH pulses. A scale bar indicates 1 mm. **(B)** Areas of BH and PSH lesions produced at given *t*_m_ and number of pulses (*p < 0.05; **p < 0.01; ***p < 0.001; ****p < 0.0001). Cross-sectioned microscopic images of the lesion produced with 10, 15, 30 and 50 pulses are plotted in Supplementary Figure S1.

With the 2 MHz HIFU transducer, *t*_m_ was set as follows: (i) 4 ms, (ii) 5 ms, (iii) 6 ms, (iv) 7 ms, (v) 8 ms and (vi) 9 ms for the PSH condition (see Table 1). Each PSH pulse had a total duration (*t*_total_) of 10 ms. At given *t*_m_ and pulse number, high-speed images of bubble dynamics (Fig. 1A-i to -vii) and lesion formation were obtained (Fig. 2A-i to A-vii). These results were then compared with BH results (BH exposure condition of a 10 ms-long 2 MHz HIFU waves with *P*_+_ = 89.1 MPa and *P*_–_ = − 14.6 MPa without pressure modulation).

**Table 1:** Pressure-modulated shockwave histotripsy (PSH) exposure conditions used in the present study.

Frequency [MHz]	Peak pressure amplitude [MPa]				Duty cycle [%]	PRF [Hz]	Pressure modulation time *t*_m_ [ms]	
	0 < *t* < *t*_m_		*t*_m_ ≤ *t* ≤ 10 ms					Pulse number
	*P*_+_ or *P*_*1*,+_	*P*_−_or *P*_*1*,−_	*P*_+_or *P*_*2*,+_	*P*_−_ or *P*_*2*,−_				
2	89.1	−14.6	29.9	−9.6	1	1	4	1, 2, 3, 4, 5, 10, 15, 30, 50
							5	1, 2, 3, 4, 5, 10, 15, 30, 50
							6	1, 2, 3, 4, 5, 10, 15, 30, 50
							7	1, 2, 3, 4, 5, 10, 15, 30, 50
							8	1, 2, 3, 4, 5, 10, 15, 30, 50
							9	1, 2, 3, 4, 5, 10, 15, 30, 50
3.5	72.4	−13.8	32.1	−9.6	1	1	4.4	1, 2, 3, 4, 5, 10, 15, 30, 50
							6	1, 2, 3, 4, 5, 10, 15, 30, 50
							7	1, 2, 3, 4, 5, 10, 15, 30, 50
							8	1, 2, 3, 4, 5, 10, 15, 30, 50
							9	1, 2, 3, 4, 5, 10, 15, 30, 50
5	69.2	−12.5	29.2	−8.6	1	1	5.4	1, 2, 3, 4, 5, 10, 15, 30, 50
							7	1, 2, 3, 4, 5, 10, 15, 30, 50
							8	1, 2, 3, 4, 5, 10, 15, 30, 50
							9	1, 2, 3, 4, 5, 10, 15, 30, 50

In the present study, it was observed that the increase in *t*_m_ proportionally correlated with the cavitation area as shown in Fig. 1. During the first 2 MHz PSH pulse with a *t*_m_ of 4 ms, the area occupied by boiling bubbles (cavitation area) was measured to be 0.146 ± 0.029 mm^2^ at the end of *t*_m_. At *t*_m_ of 5 ms, the cavitation area expanded to 0.209 ± 0.061 mm^2^ (1.43 times increase compared to *t*_m_ of 4 ms); for *t*_m_ of 6 ms, it reached 0.279 ± 0.027 mm^2^ (1.91 times increase); for *t*_m_ of 7 ms, it increased further to 0.458 ± 0.094 mm^2^ (3.14 times increase); for *t*_m_ of 8 ms, the area was 0.413 ± 0.046 mm^2^ (2.83 times increase); and for *t*_m_ of 9 ms, the area was 0.511 ± 0.060 mm^2^ (3.5 times increase). The cavitation area measured with *t*_m_ of 9 ms was approximately 20% smaller compared to that generated by the single BH pulse (0.633 ± 0.063 mm^2^). This positive relationship between *t*_m_ and cavitation area was also observed during the 2^nd^, 3^rd^, 4^th^ and 5^th^ PSH pulse (Fig. 1B). Furthermore, the cavitation area increased with increasing the number of PSH pulses at a given *t*_m_ (Fig. 1 and Supplementary Video S1). High-speed images in Fig. 1A show that the cavitation morphology varied with pulses. Some coalesced bubbles were propagated axially away from the focus by the HIFU radiation force^16 ^which induced branches or offshoots of damage from the main lesion. At the end of the *t*_m_ of 4 ms, 1, 2, 3, 4, 5, 15, 30, and 50 PSH pulses respectively resulted in the cavitation area of 0.146 ± 0.029 mm^2^, 0.272 ± 0.066 mm^2^ (1.86 times increase), 0.199 ± 0.070 mm^2^ (1.36 times increase), 0.219 ± 0.104 mm^2^ (1.5 times increase), 0.259 ± 0.043 mm^2^ (1.77 times increase), 0.195 ± 0.020 mm^2^ (1.34 times increase), 0.317 ± 0.095 mm^2^ (2.17 times increase) and 0.364 ± 0.235 mm^2^ (2.49 times increase) (Fig. 1A-ii and 1B). A similar trend was observed with *t*_m_ of 5, 6, 7, 8 and 9 ms (Fig. 1A-iii to -vii, and 1B). Linear regression analyses were performed for each pulse condition (1 to 5 pulses) to examine how cavitation area varied with increasing *t*_m_. At 2 MHz, the resulting *R*^2^ values were 0.9147 for one pulse, 0.9822 for two pulses, 0.8992 for three pulses, 0.9681 for four pulses and 0.9325 for five pulses, respectively. These consistently high *R*^2^ values support a strong positive relationship between *t*_m_ and cavitation area under all pulse conditions.

**Fig. 3:**
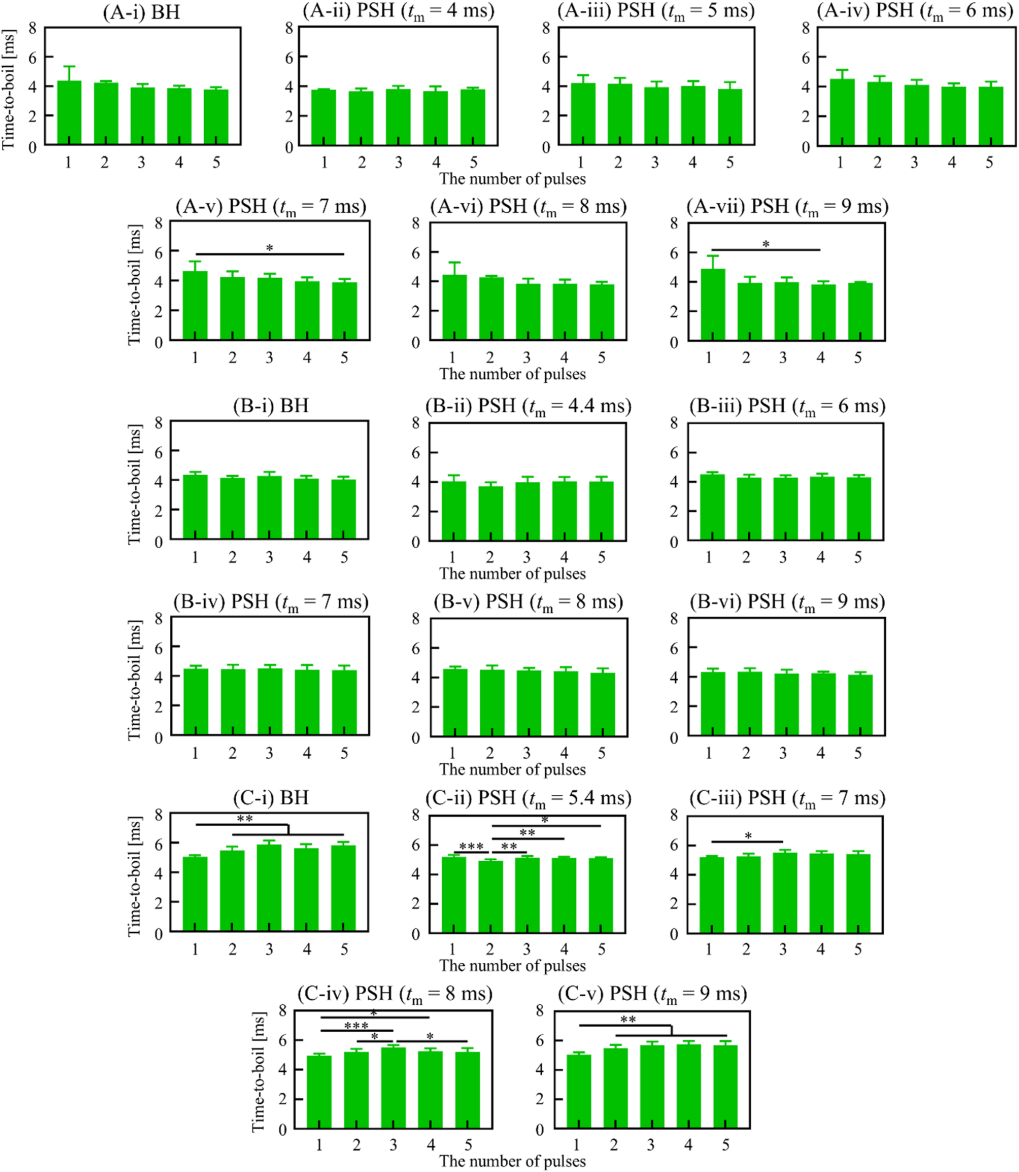
Time-to-boil measured under PSH and BH exposure conditions at different HIFU frequencies. **(A)** 2 MHz, **(B)** 3.5 MHz, and **(C)** 5 MHz. Bar graphs show the time-to-boil for the 1^st^ to 5^th^ pulse at a given *t*_m_. For 2 MHz, *t*_m_ ranges from 4 to 10 ms; for 3.5 MHz, from 4.4 to 10 ms; and for 5 MHz, from 5.4 to 10 ms. In the BH condition, *t*_total_ of 10 ms was used (*p < 0.05; **p < 0.01; ***p < 0.001). Error bars indicate standard deviation.

As the cavitation region increased with increasing the *t*_m_ and the number of PSH pulses, the size of the PSH lesion also increased accordingly (Figs. 2A-i to -vii). The lesion areas after 1, 2, 3, 4, 5, 15, 30 and 50 PSH pulses with *t*_m_ of 4 ms were measured to be 0.168 ± 0.029 mm^2^, 0.187 ± 0.050 mm^2^ (1.11 times increase), 0.233 ± 0.009 mm^2^ (1.39 times increase), 0.241 ± 0.011 mm^2^ (1.43 times increase), 0.264 ± 0.011 mm^2^ (1.57 times increase), 0.458 ± 0.043 mm^2^ (2.73 times increase), 0.506 ± 0.053 mm^2^ (3.01 times increase), 0.882 ± 0.082 mm^2^ (5.25 times increase) and 1.587 ± 0.112 mm^2^ (9.44 times increase), respectively. A similar positive relationship between *t*_m_ and lesion area was also observed, as shown in Fig. 2. Linear regression analyses were performed across different pulse conditions (1 to 5 pulses), assessing how lesion area changes with increasing *t*_m_ (5, 6, 7, 8, and 9 ms). The resulting *R*^2^ values were 0.9116 for 1 pulse, 0.9677 for 2 pulses, 0.9782 for 3 pulses, 0.9627 for 4 pulses, and 0.9640 for 5 pulses, respectively. These values indicate a strong and consistent positive correlation between *t*_m_ and lesion area across all pulse conditions. The largest PSH lesion area of 5.385 ± 0.528 mm^2^ was observed with *t*_m_ of 9 ms and 50 PSH pulses (Fig. 2B), which was comparable to the BH lesion induced with fifty 10 ms-long BH pulses (5.43 mm^2^ shown in Fig. 2B). An increase of *t*_m_ led to an increase in the PSH lesion area at a given PSH pulse number. For example, after five PSH pulses with *t*_m_ of 4 ms, the PSH lesion area was 0.264 ± 0.011 mm^2 ^which was approximately 3.21 times smaller than that produced with *t*_m_ of 9 ms (0.848 ± 0.017 mm^2^). In all cases, the sizes of the PSH lesions were, however, smaller than BH lesions, as illustrated in Fig. 2. Besides the effects of *t*_m_ and pulse number on cavitation area and lesion size, the time-to-boil at each PSH pulse (1% duty cycle (DC) and 1 Hz pulse repetition frequency (PRF)) was also measured (Fig. 3A-i and C-v). With the shortest *t*_m_ of 4 ms used, no significant statistical difference in the time-to-boil between PSH pulses was observed, whereas the time-to-boil became shortened with increasing *t*_m_.

We also noticed that the change of *t*_m_ within *t*_total_ can significantly affect the shape of a PSH lesion from an ellipsoid to a tadpole. With *t*_m_ of 4 ms, an ellipsoid PSH lesion appeared, whereas a head part of the lesion started to grow towards the HIFU transducer with increasing *t*_m_, eventually forming a well-defined tadpole shape lesion at *t*_m_ of 9 ms (Fig. 2A-ii to -vii).

### PSH using 3.5 MHz and 5 MHz HIFU transducers

**Fig. 4:**
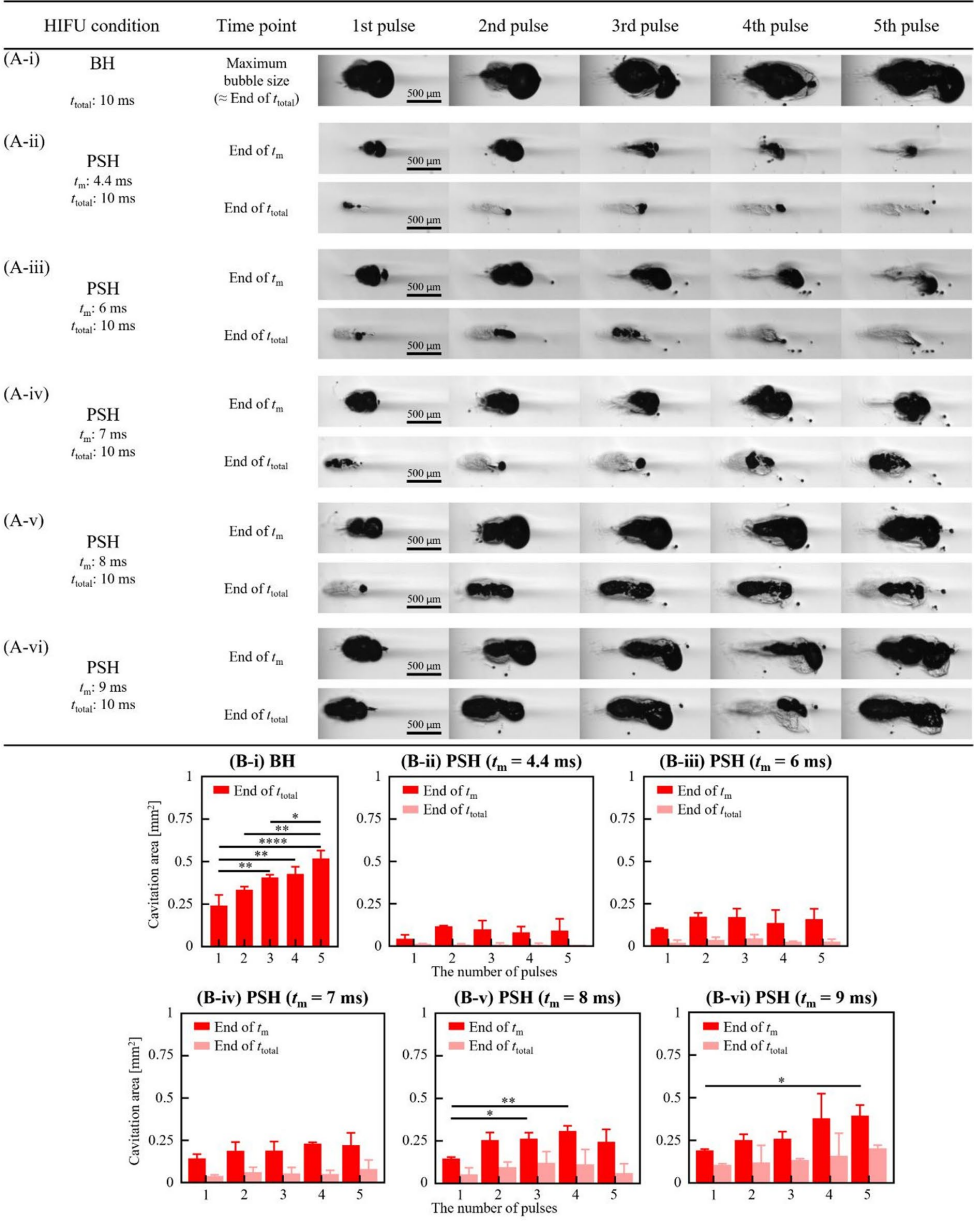
Cavitation dynamics induced by the 3.5 MHz BH and PSH exposure conditions with five pulses. High-speed camera images of bubble dynamics for **(A-i)** BH (*P*_+_ of 72.4 MPa, *P*_–_ of – 13.8 MPa) and **(A-ii to -vi)** for PSH (*P*_1,+_ of 72.4 MPa, *P*_1,–_ of – 13.8 MPa, *P*_2,+_ of 32.1 MPa and *P*_2,–_ of – 9.6 MPa) at the end of *t*_m_ and *t*_total_ at each pulse. In PSH, *t*_m_ varied from 4.4 to 9 ms within *t*_total_ of 10 ms. A DC of 1% and a pulse repetition frequency of 1 Hz were used. A scale bar indicates 500 μm. The HIFU beam propagates from left to right. A movie showing the cavitation dynamics captured during the 3.5 MHz BH and PSH exposures is available in Supplementary Video S2. **(B)** Cavitation bubble area measured at *t*_m_ and *t*_total_ with *t*_m_ of 4.4 to 9 ms and the number of pulses from 1 to 5 pulses (*p < 0.05; **p < 0.01; ***p < 0.001; ****p < 0.0001).

**Fig. 5:**
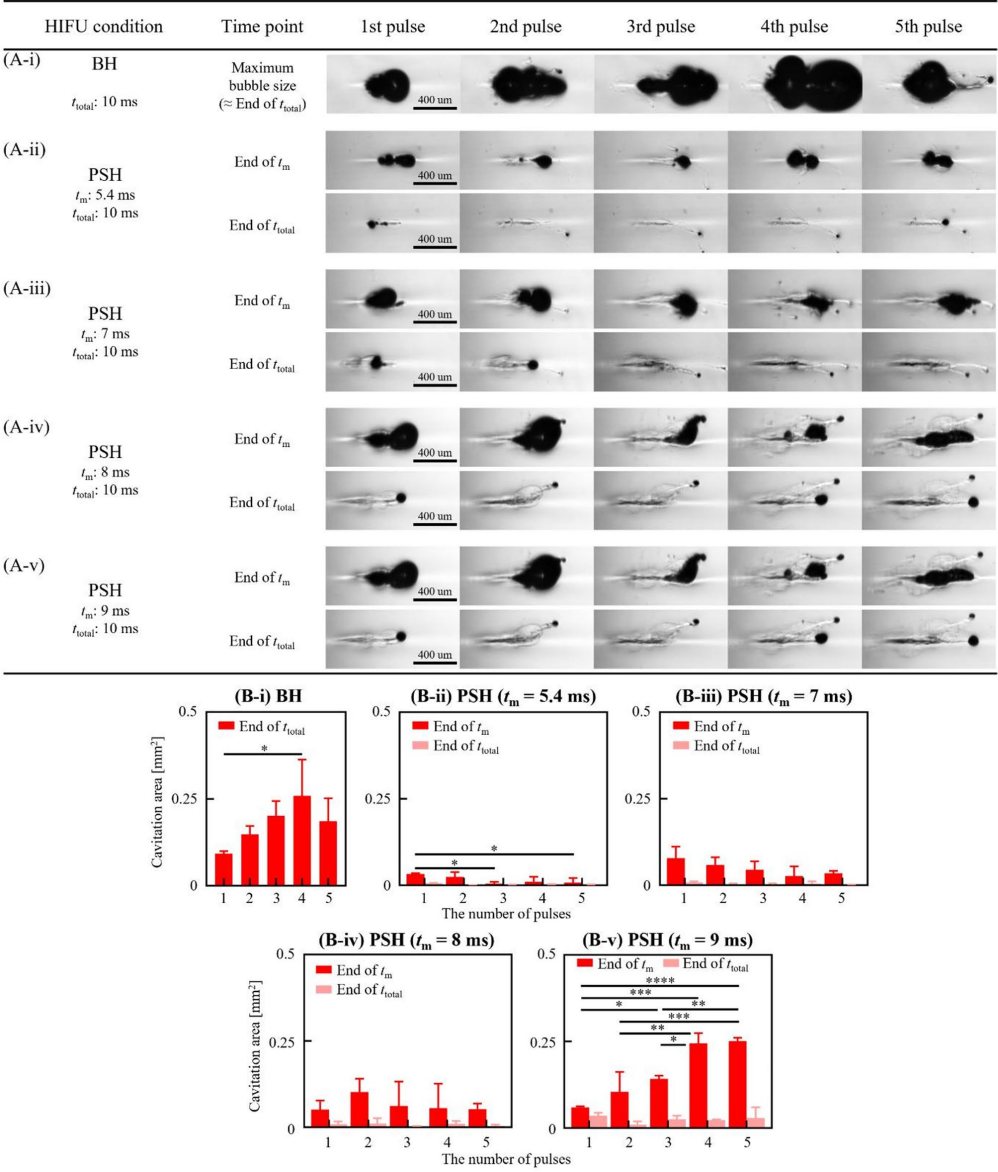
Cavitation dynamics induced by the 5 MHz BH and PSH exposure conditions with five pulses. High-speed camera images of bubble dynamics for **(A-i)** BH (*P*_+_ of 69.2 MPa, *P*_–_ of – 12.5 MPa) and **(A-ii to -v)** for PSH (*P*_1,+_ of 69.2 MPa, *P*_1,–_ of – 12.5 MPa, *P*_2,+_ of 29.2 MPa and *P*_2,–_ of – 8.6 MPa) at the end of *t*_m_ and *t*_total_ at each pulse. In PSH, *t*_m_ varied from 5.4 to 9 ms within *t*_total_ of 10 ms. A DC of 1% and a pulse repetition frequency of 1 Hz were used. A scale bar indicates 400 μm. The HIFU beam propagates from left to right. A movie showing the cavitation dynamics captured during the 5 MHz BH and PSH exposures is available in Supplementary Video S3. **(B)** Cavitation bubble area measured at *t*_m_ and *t*_total_ with *t*_m_ of 5.4 to 9 ms and the number of pulses from 1 to 5 pulses (*p < 0.05; **p < 0.01; ***p < 0.001; ****p < 0.0001).

**Fig. 6:**
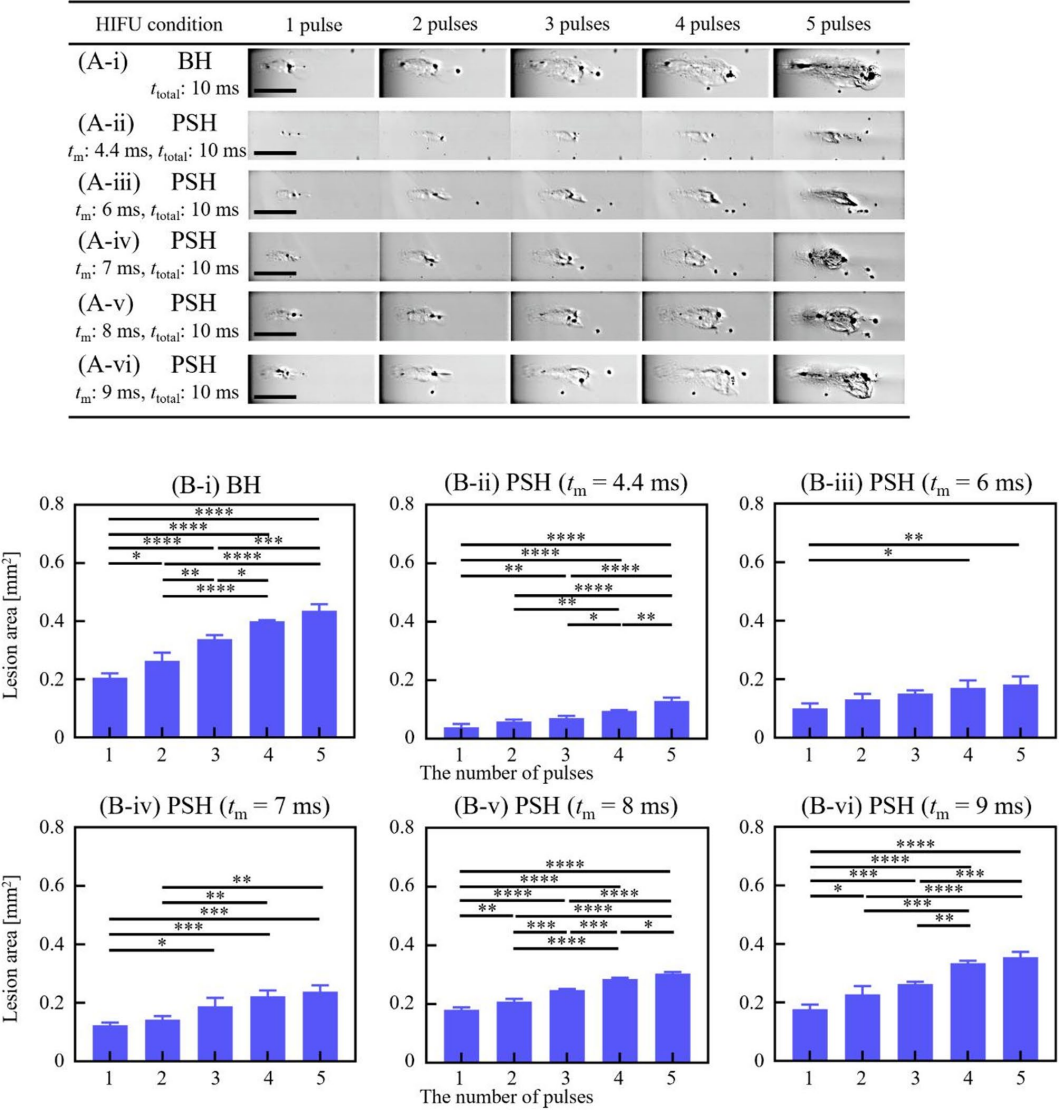
BH and PSH lesion area produced by the 3.5 MHz HIFU transducer with varying *t*_m_ and number of pulses. High-speed camera images of the lesion formation in the liver tissue phantom under various exposure conditions: **(A-i)** BH exposure conditions with a *t*_total_ of 10 ms. PSH exposure conditions with *t*_m_ of **(A-ii)** 4.4 ms, **(A-iii)** 6 ms, **(A-vi)** 7 ms, **(A-v)** 8 ms and **(A-vi)** 9 ms with the number of pulses from 1, 2, 3, 4, and 5 pulses. A scale bar indicates 500 μm. **(B)** Areas of BH and PSH lesions at given *t*_m_ and number of pulses (*p < 0.05; **p < 0.01; ***p < 0.001; ****p < 0.0001). Cross-sectioned microscopic images of the lesion produced with 10, 15, 30 and 50 pulses are plotted in Supplementary Figure S2.

**Fig. 7:**
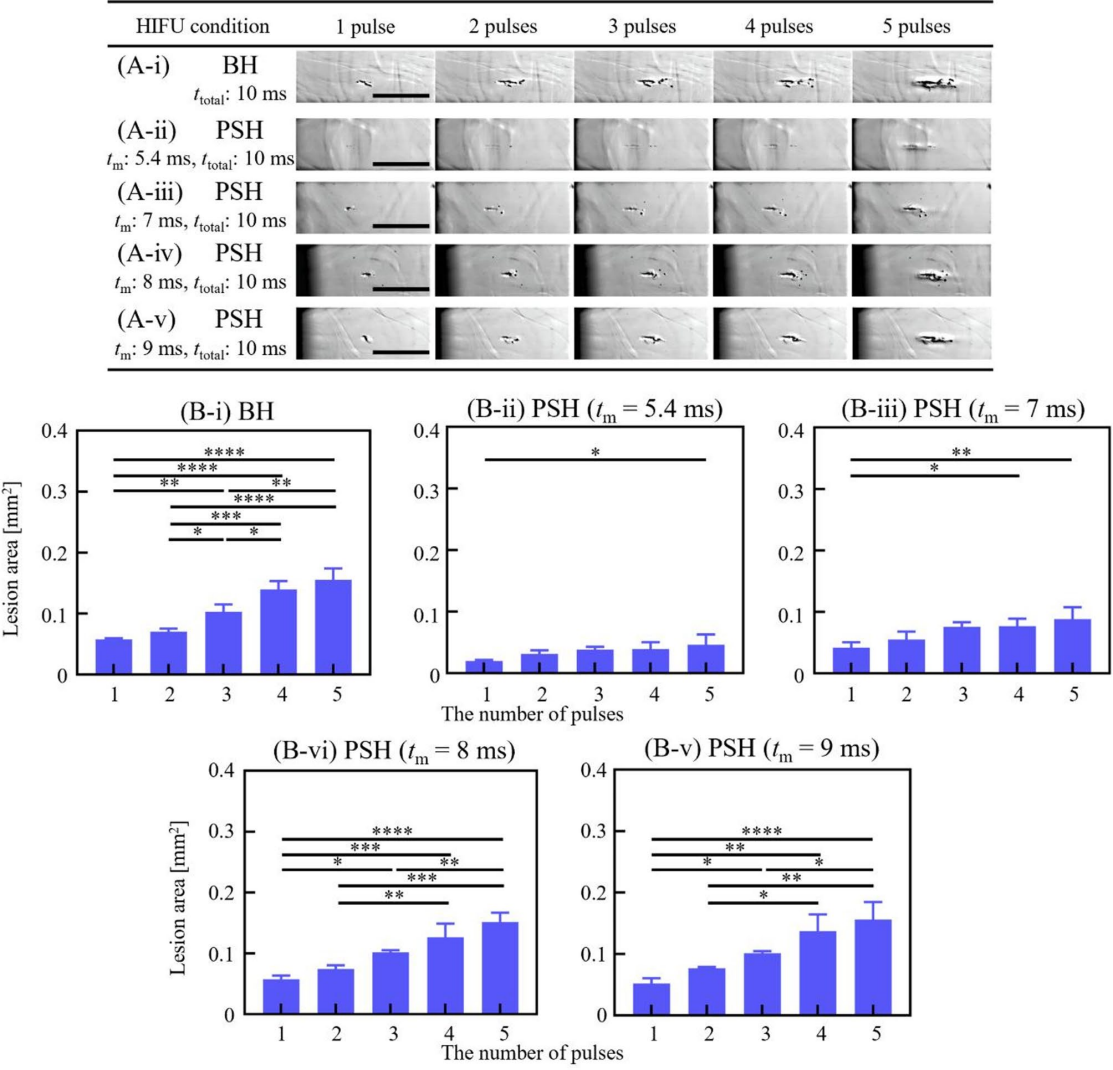
BH and PSH lesion area produced by the 5 MHz HIFU transducer with varying *t*_m_ and number of pulses. High-speed camera images of the lesion formation in the liver tissue phantom under various exposure conditions: **(A-i)** BH exposure conditions with a *t*_total_ of 10 ms. PSH exposure conditions with *t*_m_ of **(A-ii)** 5.4 ms, **(A-iii)** 7 ms, **(A-vi)** 8 ms and **(A-v)** 9 ms with the number of pulses from 1, 2, 3, 4, and 5 pulses. A scale bar indicates 500 μm. **(B)** Areas of BH and PSH lesions at given *t*_m_ and number of pulses (*p < 0.05; **p < 0.01; ***p < 0.001; ****p < 0.0001). Cross-sectioned microscopic images of the lesion produced with 10, 15, 30 and 50 pulses are plotted in Supplementary Figure S3.

**Fig. 8:**
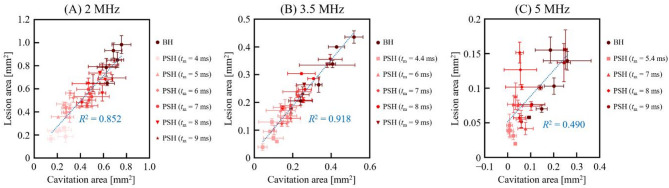
Correlation between cavitation area and lesion area under BH and PSH exposures with 1 to 5 pulses at **(A)** 2 MHz, **(B)** 3.5 MHz, and **(C)** 5 MHz. Each scatter plot illustrates the correlation between the cavitation area and the corresponding lesion area for each frequency. The blue dotted line represents the result of linear regression analysis. The *R*^2^ indicates how well the cavitation area predicts the lesion size; higher *R*^2^ values suggest a stronger linear relationship between cavitation activity and resulting tissue disruption.

The same analyses conducted with the 2 MHz HIFU source were carried out using the 3.5 MHz and 5 MHz HIFU sources. Cavitation bubble dynamics and lesion formations were investigated with various 3.5 MHz and 5 MHz PSH exposure conditions (Table 1). For the 3.5 MHz HIFU source, *t*_m_ was as follows: 4.4, 6, 7, 8 and 9 ms, and *t*_m_ for the 5 MHz HIFU transducer varied as 5.4, 7, 8, and 9 ms. Since the shockwave heating is dominant by the shock amplitude, this slight increase in minimum *t*_m_ with frequency observed in the present study is primarily due to the lower peak pressure amplitudes employed in the 3.5 MHz and 5 MHz transducers compared to those used in the 2 MHz transducer. Results on cavitation area and lesion size obtained with the 3.5 and 5 MHz PSH approaches were compared with those obtained with the 3.5 MHz (i.e., 10 ms pulse length, *P*_+_ = 72.4 MPa and *P*_–_ = − 13.8 MPa without pressure modulation) and 5 MHz (10 ms pulse length, *P*_+_ = 69.2 MPa and *P*_–_ = − 12.5 MPa without pressure modulation) BH exposure conditions without pressure modulation (Figs. 4, 5 and 6, and 7; Supplementary Videos S2 and S3). Similar to the previous observations shown in Figs. 1 and 2, the cavitation area (Figs. 4 and 5) as well as the size of a PSH lesion (Figs. 6 and 7) gradually increased with increasing *t*_m_ and pulse number. When comparing the 3.5 and 5 MHz PSH results, the 5 MHz PSH exposure (*t*_m_ = 5.4 ms, 5 pulses) was found to be more effective in producing a smaller cavitation area compared to the 3.5 MHz PSH exposure (*t*_m_ = 4.4 ms, 5 pulses) with measured maximum cavitation areas of 0.092 ± 0.069 mm^2^ and 0.008 ± 0.013 mm^2 ^respectively (Figs. 4 and 5). This corresponds to approximately an 11.5-fold reduction in cavitation area at 5 MHz compared to 3.5 MHz under these conditions. Throughout the 2, 3.5 and 5 MHz PSH experiments, no thermal damage, which would manifest itself as an opaque lesion in the tissue phantom^13^was observed in the tissue phantoms, suggesting that the extent of mechanical damage produced by the boiling bubbles was likely larger than the extent of shockwave heated region. The largest PSH lesion was observed with the 2 MHz HIFU transducer. To further elucidate the relationship between cavitation dynamics and resulting lesion formation, quantitative comparisons were conducted between the cavitation area and lesion area across all pulse conditions (1 to 5 pulses) for the 2, 3.5, and 5 MHz PSH exposures. Scatter plots with linear regression lines were generated, and the *R*^2^ was calculated to assess the strength of correlation. The *R*^2^ values were found to be 0.852, 0.918, and 0.490 for the 2, 3.5, and 5 MHz, respectively, indicating a strong correlation at 2 and 3.5 MHz, and a moderate correlation at 5 MHz (Fig. 8).

### PCD signals during the 2, 3.5 and 5 MHz PSH exposures

**Fig. 9:**
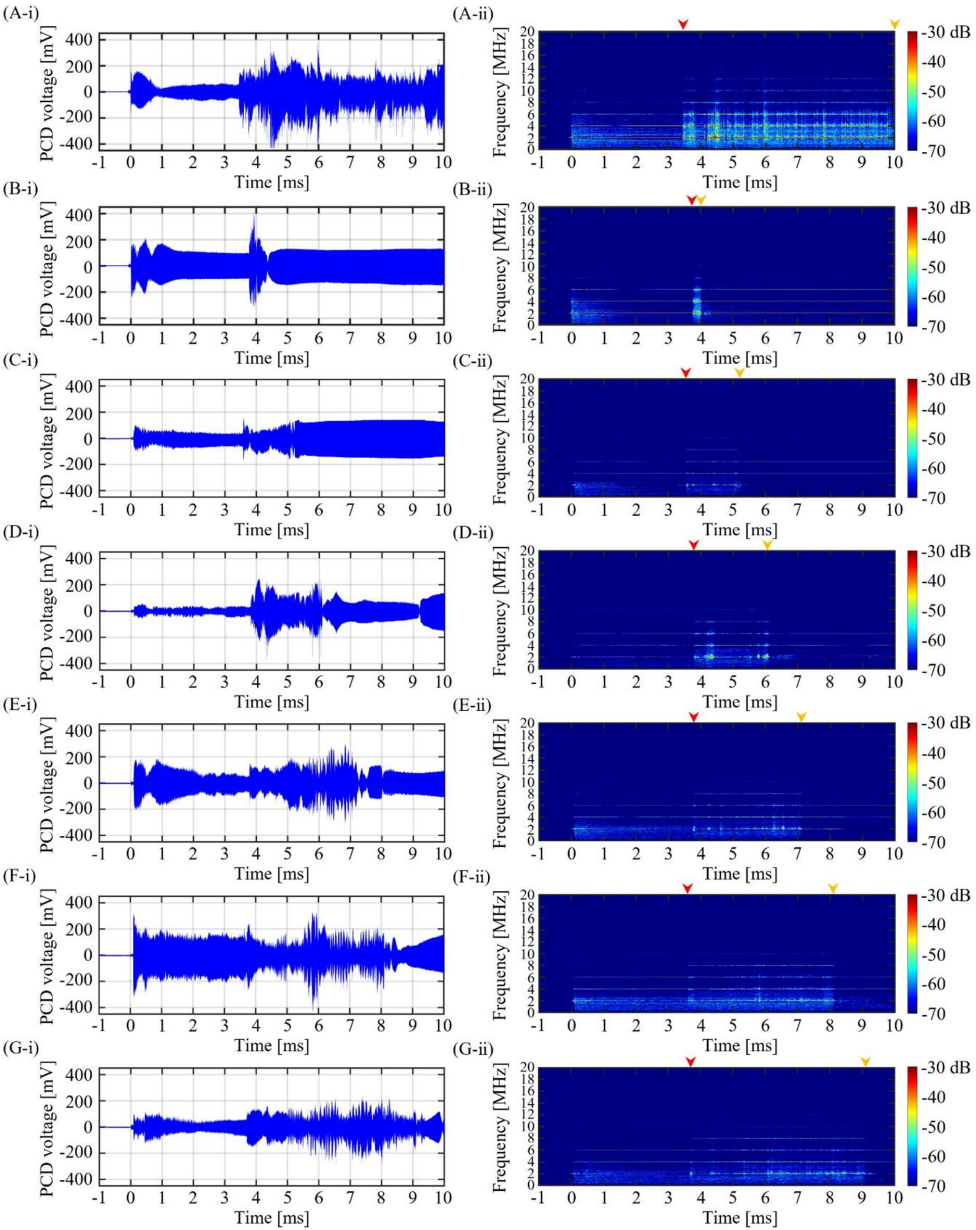
PCD signal during the 2 MHz BH or PSH exposure conditions. **(A-i)** Plot of PCD voltage versus time during the first BH pulse (*t*_total_ of 10 ms). **(A-ii)** The corresponding spectrogram of (A-i). PCD voltage versus time plot and the corresponding spectrogram during the first PSH with varying *t*_m_ at **(B)** 4 ms, **(C)** 5 ms, **(D)** 6 ms, **(E)** 7 ms, **(F)** 8 ms and **(G)** 9 ms. In each spectrogram, red and orange arrows above the plots indicate the onset and end of the significant broadband emission region, respectively.

**Fig. 10:**
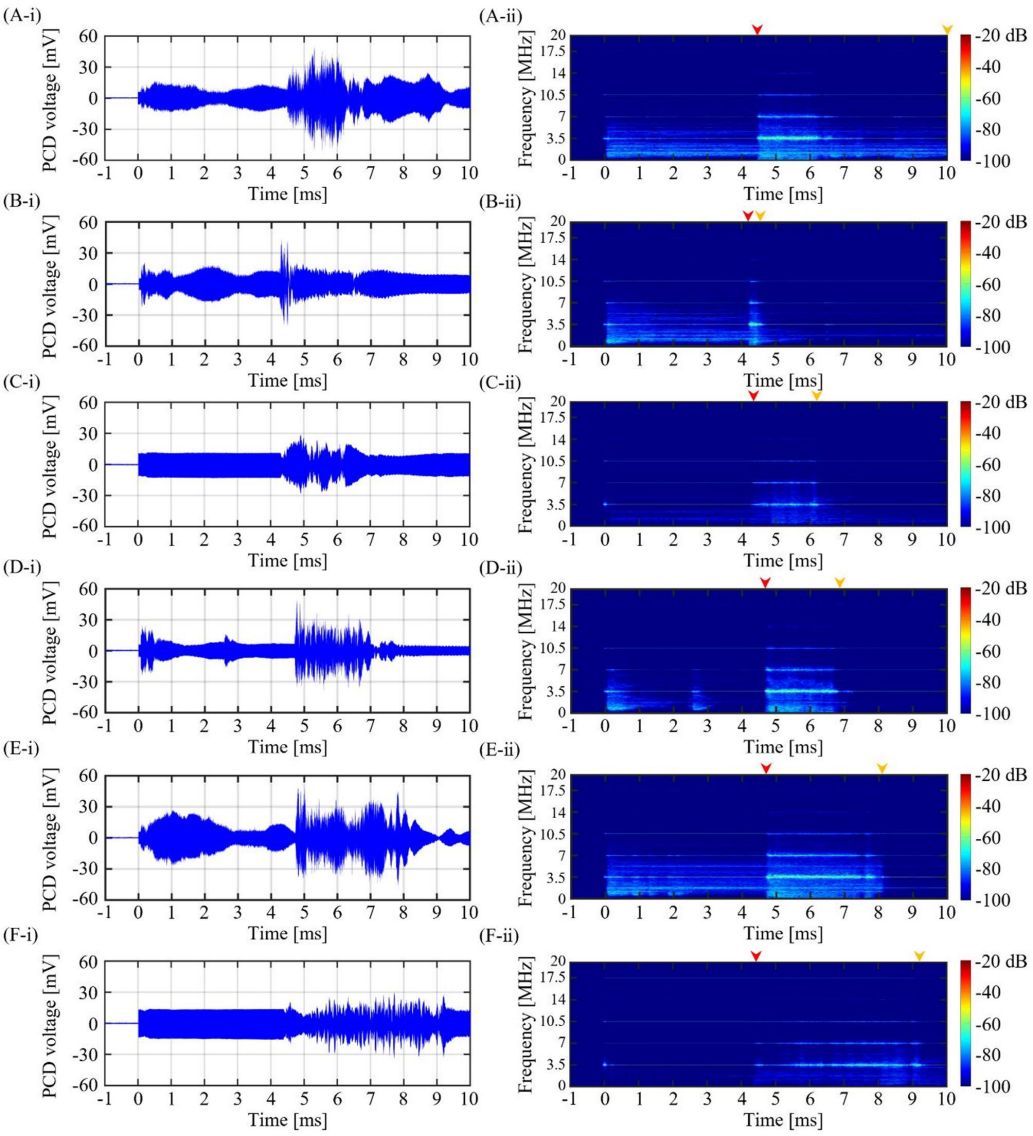
PCD signal during the 3.5 MHz BH or PSH exposure conditions. **(A-i)** Plot of PCD voltage versus time during the first BH pulse (*t*_total_ of 10 ms). **(A-ii)** The corresponding spectrogram of (A-i). PCD voltage versus time plot and the corresponding spectrogram during the first PSH with varying *t*_m_ at **(B)** 4.4 ms, **(C)** 6 ms, **(D)** 7 ms, **(E)** 8 ms and **(F)** 9 ms. In each spectrogram, red and orange arrows above the plots indicate the onset and end of the significant broadband emission region, respectively.

**Fig. 11:**
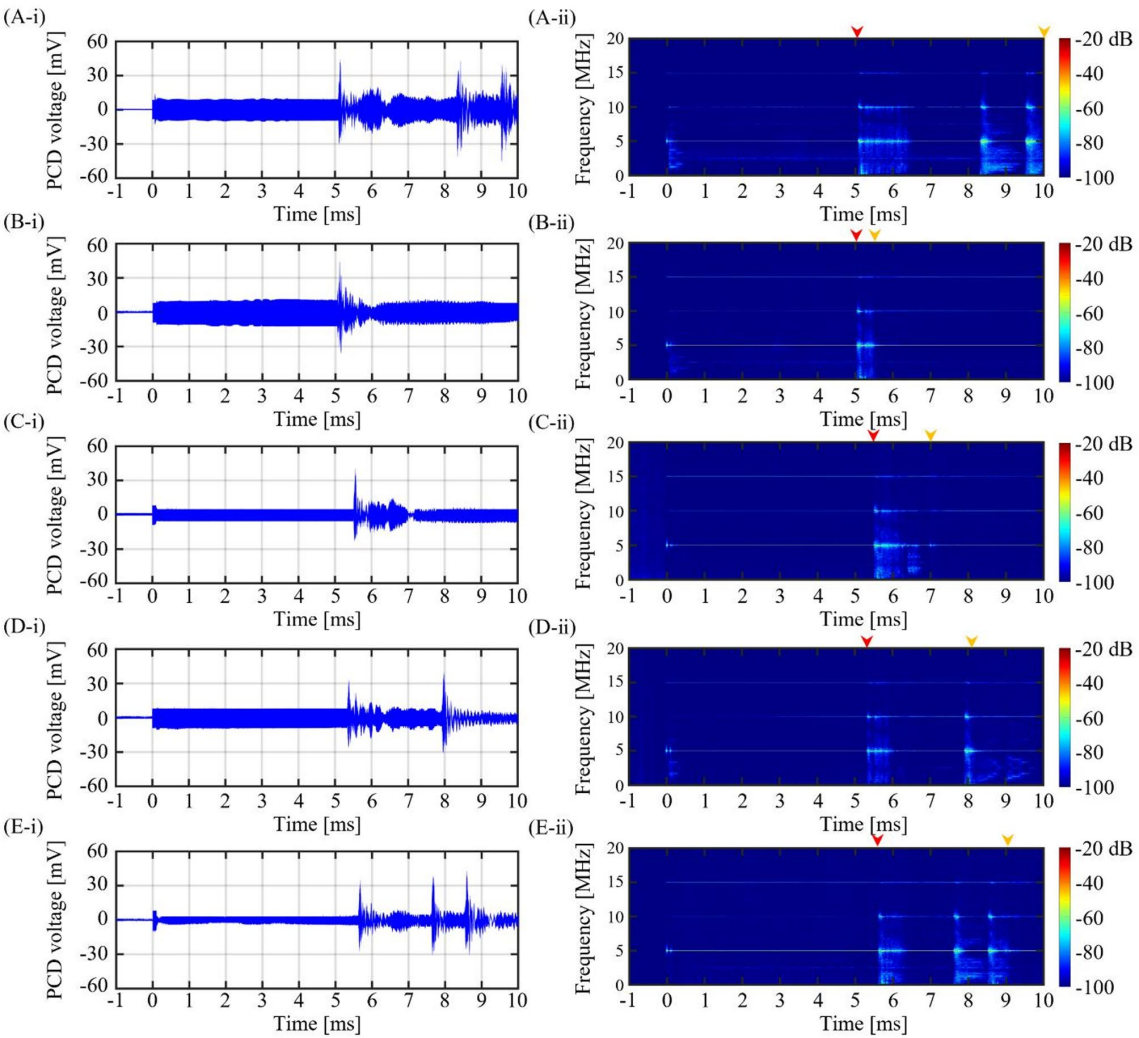
PCD signal during the 5 MHz BH or PSH exposure conditions. **(A-i)** Plot of PCD voltage versus time during the first BH pulse (*t*_total_ of 10 ms). **(A-ii)** The corresponding spectrogram of (A-i). PCD voltage versus time plot and the corresponding spectrogram during the first PSH with varying *t*_m_ at **(B)** 5.4 ms, **(C)** 7 ms, **(D)** 8 ms and **(E)** 9 ms. In each spectrogram, red and orange arrows above the plots indicate the onset and end of the significant broadband emission region, respectively.

Under a given PSH exposure condition, PCD voltage signals were recorded during the first PSH pulse and were analysed in spectrogram, as shown in Figs. 9, 10 and 11. With the 2 MHz BH exposure condition (i.e., no pressure modulation), a high-amplitude spike was observed in the time domain when the initial boiling bubble formed at 3.45 ms (i.e., time to form a boiling bubble) (Fig. 9A-i). The corresponding spectrogram showed higher order harmonics of 2 MHz. After the observation of the multiple harmonic components, significant broadband emissions were observed, which were maintained until the end of the BH exposure (Fig. 9A-ii). These broadband emissions were likely related to the shock scattering effect (i.e., formation of inertial bubble clouds)^6,15^. Under the 2 MHz PSH exposure, spikes of high amplitude of PCD voltage were observed when boiling bubbles were produced at 3.75 ± 0.05 ms (Fig. 9B-i to G-i) along with the occurrence of the higher order multiple harmonics in the corresponding spectrograms (Fig. 9B-ii to G-ii). Broadband emissions appeared and persisted between the time to form a boiling bubble (i.e., time-to-boil) and *t*_m_. After *t*_m_, higher order multiple harmonics were only observed without significant broadband noise (Fig. 9B-ii to G-ii). The same experimental observations described above were also obtained under the 3.5 and 5 MHz HIFU sources (i.e., a high-amplitude spike appeared when a boiling bubble was produced. Significant broadband emissions disappeared after *t*_m_) (Figs. 10 and 11).

## Discussion

In the present study, we investigated the effects of variations of *t*_m_ and number of PSH pulses on bubble dynamics and lesion formation including its size and shape changes. Our experimental results clearly show that bubble dynamics and lesion formation can be effectively controlled by simply adjusting *t*_m_ within a single PSH pulse. For instance, when *t*_m_ was close to the time-to-boil (i.e., the time when a boiling bubble forms by shockwave heating), shockwave heating-induced boiling bubbles were coalesced and sustained throughout the PSH exposure without the significant shock scattering effect (Figs. 1A-ii to -vii, 4 A-ii to -vi, 5 A-ii to -v and 12 C). Since the pressure magnitude of the backscattered acoustic field was likely to be below the intrinsic cavitation threshold of – 28 MPa with *P*_2,*+*_ and *P*_2,–_^16^, there was no significant broadband emission appearing in the spectrograms after *t*_m_ (Figs. 9B-ii to G-ii, 10B-ii to F-ii and 11B-ii to E-ii). Boiling bubbles mainly contributed to mechanical damage. The shape of a PSH lesion was, therefore, an ellipsoid (Figs. 2A-ii, 6 A-ii and 7 A-ii). This oval shaped PSH lesion was mainly produced by the generation of shear stresses around boiling bubbles^6^. A further increase in *t*_m_ beyond the time-to-boil increased the cavitation area due to the initiation of the shock scattering effect (Figs. 1B, 4B and 5B). We observed that *t*_m_ of 9 ms eventually induced a larger cavitation area than that induced by *t*_m_ of 4 ms, whose size was comparable to that produced by the 10 ms-long BH pulses (Fig. 1). The PSH lesion size also increased accordingly with increasing *t*_m_ (Figs. 2B and 6B, and 7B). Because the shock scattering effect can appear with the use of a longer *t*_m_ than the time-to-boil, the gradual increase of *t*_m_ can result in the production of a PSH lesion with a more tadpole-like (Figs. 2A-i to -vii, 6 A-i to -vi and 7 A-i to -v), which is the typical shape of a BH lesion^13,15^. It has also been reported that the size of a BH lesion can be changed with varying the BH pulse length (as opposed to changing *t*_m_ in PSH approach). In BH, the explosion of a boiling bubble and its further interaction with shocks can cause mechanical tissue fractionation. A BH pulse length therefore needs to be longer than the time-to-boil^11,13^. Ponomarchuk et al.^20^ experimentally showed that 2 ms-long BH pulses (1.5 MHz, *P*_+_ of 120 MPa, *P*_–_ of − 17 MPa) induced a smaller tadpole shaped BH lesion than 10 ms-long BH pulses. Because of the appearance of the shock scattering effect involved in BH, the overall shape of a BH lesion remained unchanged. To further shorten the time-to-boil in BH at a given driving frequency, higher peak pressure magnitudes should, however, be applied.

In addition to the effects of the change of *t*_m_, the sizes of cavitation area and PSH lesion were also found to be dependent on the number of PSH pulses. The cavitation area increased with the number of PSH pulses at a given *t*_m_, as observed in Fig. 1. As the number of PSH pulses increases, the bubble grows as it liquefies more of the tissue resulting in an increase in the cavitation area. This, however, depends upon the mechanical property of the target tissue (e.g., stiffness). Specifically, when *t*_m_ was around the time-to-boil, the maximum cavitation areas generated by 2, 3.5, and 5 MHz PSH with 5 pulses were 0.458, 0.126, and 0.029 mm^2 ^respectively. These values were several times smaller than those observed with 50 PSH pulses, which were 1.587, 0.386, and 0.175 mm^2^ at 2, 3.5 and 5 MHz PSH, respectively (see Figs. 2B, S2B, and S3B). The 5-pulse condition was selected to better illustrate the dependence of cavitation area on the number of PSH pulses. Under the exposure of multiple PSH pulses, accelerated boiling with later PSH pulses can likely appear, which is primary due to buildup heat and not to the presence of residual cavitation nuclei^13^. This accelerated boiling can then contribute to enlarging the PSH lesion size. This explanation is plausible since the PSH method is also based upon the shockwave heating effect^16^ which is one of the main mechanisms involved in BH. During our experiments, some discontinuous damage appeared within the HIFU focal zone at a relatively low number of PSH pulses (Fig. 2A, Supplementary Fig. S1). After 50 PSH pulses, more homogenous damage distribution was observed. This can imply that, for achieving a certain level of precision of tissue destruction, a certain number of PSH pulses should be employed to result in a complete lesion fractionation with homogeneous damage distribution. With similar purposes, in BH, fifty BH pulses are typically applied for complete mechanical tissue fractionation.

In BH, the time-to-boil essentially decreases with BH pulses because of an accumulation of heat between pulses^13^. In the present study, when the *t*_m_ was at the time-to-boil, there was no significant difference on the onset time of a boiling bubble formation between PSH pulses (Fig. 3). The percentage difference in the time-to-boil between the 1^st^ and 5^th^ PSH pulses was 1.048 ± 0.042% with the 2 MHz PSH (*t*_m_ of 4 ms), 0.576 ± 0.180% with the 3.5 MHz PSH (*t*_m_ of 4.4 ms), and 1.758 ± 0.039% with the 5 MHz PSH (*t*_m_ of 5.4 ms). However, with an increase in *t*_m_, a notable discrepancy on the time-to-boil was observed, particularly at 2 MHz. In case of the 2 MHz PSH, the reduction percentage of the time-to-boil was − 9.671 ± 0.233%, − 11.847 ± 0.195%, − 16.308 ± 0.165%, − 14.727 ± 0.195% and − 19.653 ± 0.156% with increasing *t*_m_ from 4 ms to 5, 6, 7, 8 and 9 ms, respectively. This is likely because that an increase in *t*_m_ would result in an increase in an accumulation of heat between pulses, whereby decreasing the time-to-boil. For the highest frequency transducer (5 MHz), an opposite trend was, however, observed. At higher *t*_m_ values, the time-to-boil slightly increased with pulses. This might be due to the potential reduction of *P*_1,+_ and *P*_1,−_ resulted from scattering of incoming waves by residual small bubbles (scattering increases with frequency), limiting thermal buildup between PSH pulses at 5 MHz. This trend was also observed with the 5 MHz BH exposure condition, shown in Fig. 3(C-i). These explanations can also be applied to the moderate correlation observed between the cavitation area and lesion at 5 MHz (Fig. 8C). In contrast to BH, since PSH employs lower *P*_+_ and *P*_−_ from *t*_m_ to *t*_total_ (i.e., *P*_2,+_ < *P*_1,+_ and *P*_2,–_ < *P*_1,–_), a relatively lower temperature increase at the HIFU focus than BH would be expected, suggesting that PSH could (a) further reduce the potential risk of thermal damage and (b) be used with higher than 1 Hz PRF which is typically used for BH exposure. The use of higher PRF would be beneficial for enhancing mechanical tissue fractionation process; however, this would also increase the risk of thermal heating, which warrants further study.

Whilst the present study was conducted in the liver tissue phantom, our in vitro results can provide important insights for clinical translation. The liver phantom was used to reflect the potential application of PSH to the treatment of liver diseases (e.g., solid tumours, liver fibrosis etc.), where precise lesion control is critical due to the presence of a complex vascular network. The changes of lesion size and shape by simply adjusting *t*_m_ and the number of pulses suggest that PSH could be further tailored to selectively destroy target tumour region or margin region whilst minimising mechanical damage to surrounding blood vessels and tissues. Nevertheless, our experiments conducted in the liver tissue phantoms had inherent limitations compared to in vivo scenarios such as blood perfusion, tissue heterogeneity and physiological motion. These may affect cavitation thresholds, heat dissipation and PSH lesion morphology. In addition, in vivo human liver tissue exhibits considerably more biological variability due to individual differences in fat content, fibrosis, perfusion, and other disease-related changes. These factors may also affect acoustic propagation, cavitation thresholds and lesion formation. For example, sound speed in fatty liver has been reported to drop to approximately 1525 m/s, whilst attenuation coefficients may exceed 1.2 dB/cm·MHz in diseased tissue. In line with this, Joung and Heo et al.^5^ showed that the size of histotripsy lesion produced in fibrotic liver tissue was smaller than that induced in normal liver tissue in rat’s liver in vivo at a given exposure condition, highlighting how pathological changes in tissue mechanical and acoustic properties can possibly affect therapeutic efficacy. Therefore, these should be carefully considered when designing a PSH pulsing protocol for targeting different pathological conditions of the liver.

## Conclusion

In this study, we investigated the effects of the variations of the *t*_m_ and number of pulses on bubble dynamics and lesion formation. The results clearly showed that the *t*_m_ in PSH is the primary parameter that can determine the magnitude and extent of mechanical damage and shape of a PSH lesion. An increase of *t*_m_ beyond time-to-boil in PSH can lead to the shock scattering effect, forming a larger lesion with the shape of a tadpole, and vice versa, a decrease in *t*_m_ can minimise or eliminate the shock scattering effect, producing a smaller PSH lesion in the form of an ellipsoid. These results can suggest that PSH can be a promising treatment option for treating solid tumours with different sizes and locations. For instance, a large tumor could be treated with PSH with a long *t*_m_, and subsequently, a shorter *t*_m_ could then be used to treat the residual tumour at the margin using the same HIFU transducer. Further investigations with in vivo models are needed to confirm and further understand the impact of change of *t*_m_ on lesion formation.

## Methods

### Experimental setup

**Fig. 12:**
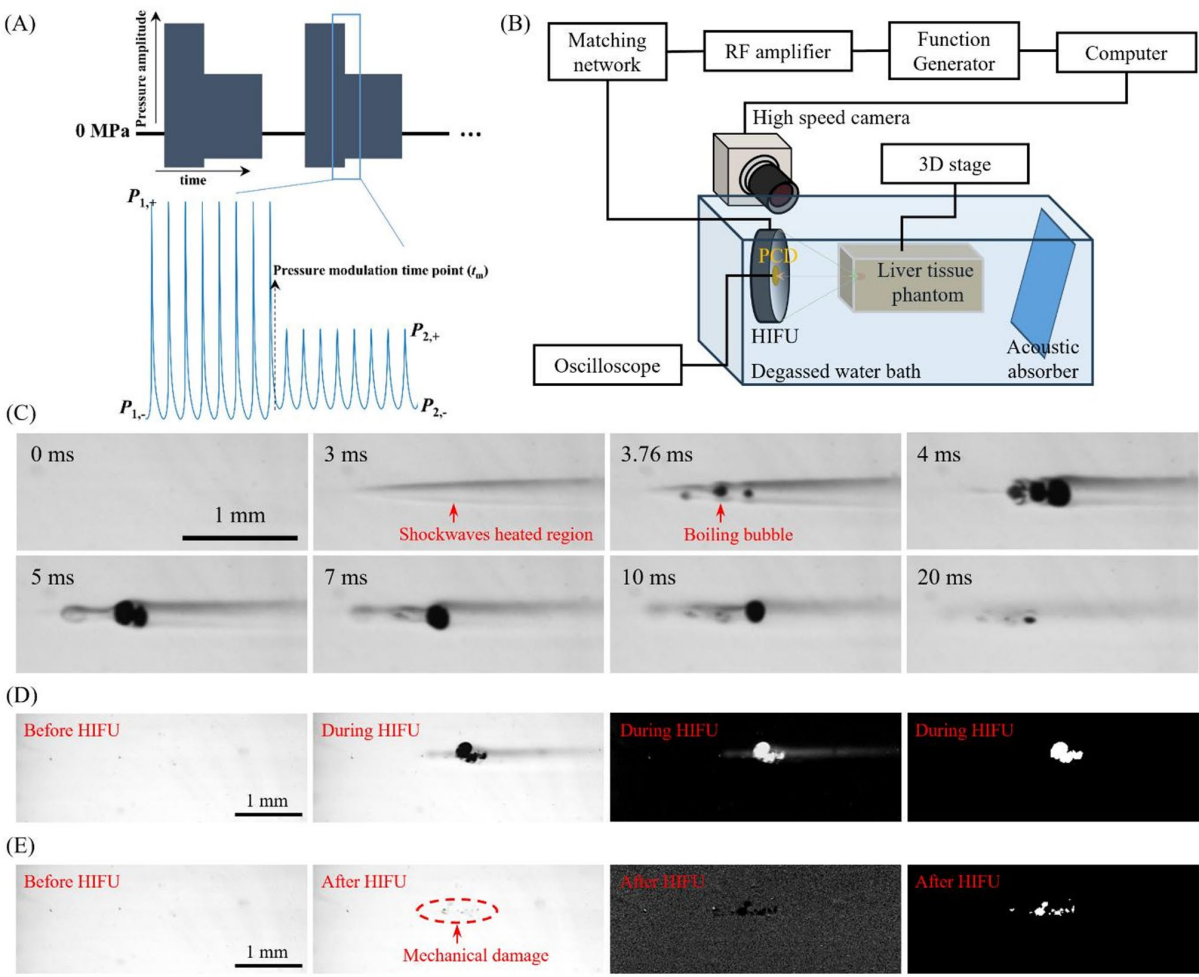
Schematic diagrams of **(A)** the acoustic waveforms and **(B)** the experimental setup used for pressure-modulated shockwave histotripsy (PSH). **(C)** A series of representative high-speed images of the cavitation bubble dynamics induced with a single 2 MHz PSH pulse with a *t*_m_ of 4 ms, *t*_total_ of 10 ms, *P*_1,+_ of 89.1 MPa, *P*_1,–_ of – 14.6 MPa, *P*_2,+_ of 29.9 MPa and *P*_2,–_ of – 9.6 MPa. Boiling bubbles were produced within the localised shockwaves heated region. Detection of **(D)** cavitation area and **(E)** PSH lesion from the high-speed images. The red arrow indicates the mechanical damage after the exposure.

To investigate the potential effects of changes of PSH exposure conditions on bubble dynamics and lesion formation, the following experimental setup was employed (Fig. 12B). The setup consists of a single element bowl-shaped 2, 3.5 or 5 MHz HIFU transducer (#H148, #SU-107, #SU-108, Sonic Concepts Inc., WA, USA) placed in a Perspex bath filled with degassed and deionised water. These HIFU transducers were the same transducers used in the previous PSH proof-of-concept study^16^ and were previously characterised using a calibrated needle hydrophone in water under linear wave propagation with a reported calibration uncertainty of approximately ± 1 dB in the 1–15 MHz frequency range. The potential sources of uncertainty were the uncertainty in the calibration of the reference hydrophone, temperature variation, source stability, digitizer error and positional repeatability. Characteristics of the HIFU transducers employed in the present study are shown in Table 2. The HIFU sources were driven by an arbitrary wave generator (33600 A, Agilent, CA, USA) and a radiofrequency power amplifier (1040 L, ENI, NY, USA). A 20 mm diameter PCD transducer (64 mm radius of curvature, bandwidth of 10 kHz to 15 MHz, Y107, Sonic Concepts Inc., WA, USA) was placed in the centre of the 2 MHz HIFU source. The PCD and the 2 MHz HIFU transducers were aligned coaxially and confocally. For the 3.5 MHz or 5 MHz HIFU transducer (33 mm in diameter and 35 mm radius of curvature, no central opening to align the PCD coaxially), the PCD transducer was positioned perpendicular to the focal axis of the HIFU transducer and aligned confocally. During the experiments, cavitation emission signals received by the PCD transducer were recorded by a digital oscilloscope (WaveSurfer 3054z, Teledyne LeCroy, NY, USA) at a sampling rate of 4 GS/s.

**Table 2:** Characteristics of the three different HIFU transducers employed in the present study.

Transducer model	Frequency	Aperture size	Radius of curvature	Axial full width half maximum (FWHM) pressure dimension in water*	Lateral full width half maximum (FWHM) pressure dimension in water*
H148	2 MHz	64 mm	63.2 mm	7.25 mm	0.89 mm
SU-107	3.5 MHz	33 mm	35 mm	3.5 mm	0.45 mm
SU-108	5.0 MHz	33 mm	35 mm	2.65 mm	0.32 mm

*These values were calibrated and measured in previous PSH study^16^.

In the present study, we used tissue phantom (10 cm × 2 cm × 2 cm) which has similar acoustic and thermal properties to those of normal liver tissue (sound speed of 1544 m/s, density of 1044 kg/m^3^, absorption coefficient of 15 dB/m/MHz, coefficient of nonlinear of 4.0, specific heat capacity per unit volume of 5.3 × 10^6^ J/m^3^/^o^C)^3,13,21^. The phantom was fabricated following the protocol described in a previous study^15^. During our experiments, a manual 3D positioning stage (TS-ONE stage & Optics, Republic of Korea) was used to control the position of the phantom, and the HIFU focus was positioned 5 mm below the surface of the phantom. Furthermore, a high-speed camera operating at 100,000 frames per second (320 × 120 resolution, 21.5 μm per pixel, Phantom VEO1010L, Vision Research, NJ, USA) was used to observe cavitation dynamics at a given PSH exposure condition. Illumination for high-speed imaging was provided by a halogen lamp positioned outside the water tank, which allowed clear visualisation of both cavitation activity and lesion formation.

### PSH exposure conditions and pressure modulation time point

The same PSH exposure conditions (*P*_*+*_ and *P*_–_ at *t* < *t*_m_ or *P*_1,*+*_ and *P*_1,–_, and *P*_*+*_ and *P*_–_ at *t* ≥ *t*_m_ or *P*_2,*+*_ and *P*_2,–_) used in our previous PSH study^17^ were applied in the present study (Fig. 12A). For the 2 MHz HIFU source, *P*_+_ and *P*_–_ were at 89.1 MPa and − 14.6 MPa from *t* = 0 to *t*_m_ (0 ≤ *t* < *t*_m_). Subsequently, *P*_+_ of 29.9 MPa and *P*_–_ of − 9.6 MPa were employed from *t*_m_ to the end of the total pulse duration (*t*_m_ ≤ *t* ≤ *t*_total_). With the 3.5 MHz HIFU source, *P*_*+*_ and *P*_–_ at 0 ≤ *t* < *t*_m_ and *t*_m_ ≤ *t* ≤ *t*_total_ were respectively 72.4 MPa; − 13.8 MPa, and 32.1 MPa; − 9.6 MPa. For the 5 MHz HIFU source, *P*_+_ of 69.2 MPa; *P*_–_ of − 12.5 MPa at 0 ≤ *t* < *t*_m_ and *P*_+_ of 29.2 MPa and *P*_–_ of − 8.6 MPa at *t*_m_ ≤ *t* ≤ *t*_total_ were employed. For simplicity, *t*_total_ was kept constant at 10 ms. These acoustic pressure values were obtained by solving the Khokhlov-Zabolotskaya-Kuznetsov (KZK) nonlinear wave equation^6^.

The minimum required *t*_m_ for each HIFU transducer was initially determined according to the time at which a boiling vapour bubble formed at the HIFU focus in the tissue phantom at a given *P*_1,*+*_ and *P*_1,–_, which was considered as time-to-boil. With the 2 MHz HIFU transducer (*P*_+_ = 89.1 MPa and *P*_–_ = − 14.6 MPa), boiling bubbles were observed at 3.75 ± 0.05 ms (mean ± standard deviations with *n* = 5). Similarly, with the 3.5 MHz HIFU transducer (*P*_+_ = 72.4 MPa and *P*_–_ = − 13.8 MPa), boiling bubbles formed at 4.39 ± 0.28 ms (*n* = 36), and boiling bubbles appeared at 5.09 ± 0.16 ms (*n* = 32) with the 5 MHz HIFU transducer (*P*_+_ = 69.2 MPa; *P*_–_ = − 12.5 MPa). These relatively small standard deviations observed in the repeated experiments showed small phantom-to-phantom variation of physical property (i.e., high reproducibility). From these experimental observations, the minimum required *t*_m_ was chosen as follows: 4, 4.4 and 5.4 ms for the 2, 3.5 and 5 MHz HIFU transducers, respectively. To investigate the effect of the change of *t*_m_ on bubble dynamics and lesion formation, the *t*_m_ varied from the minimum *t*_m_ to 9 ms. In addition to this, the number of PSH pulses changed from 1 to 50 at a given *t*_m_ with 1% DC and 1 Hz PRF. Table 1 shows the summary of the PSH exposure conditions. Figure 12C illustrates a sequence of high-speed camera images capturing the bubble dynamics under the single 2 MHz PSH pulse (*t*_m_ at 4 ms, *t*_total_ of 10 ms, *P*_*+*_ = 89.1 MPa and *P*_–_ = − 14.6 MPa at *t* < *t*_m_, and *P*_+_ = 29.9 MPa and *P*_–_ = − 9.6 MPa at *t*_m_ ≤ *t* ≤ *t*_total_). As can be seen, the shockwave heated region in the HIFU focus was observed at 3 ms followed by the initial formation of the boiling bubbles at 3.76 ms. The bubbles started to coalesce at *t*_m_ of 4 ms, forming a coalescent bubble with a size of approximately 0.26 mm. This enlarged and coalesced bubble was maintained until the end of the PSH pulse. Similar experimental observations on bubble dynamics were also observed with the 3.5 and 5 MHz frequencies^16^.

### Analysis method for measuring the areas of cavitation and PSH lesion

The high-speed camera images were post-processed using ImageJ open software (National Institutes of Health, MD, USA) to quantify and analyse the areas of cavitation and PSH lesion. As shown in Fig. 12 (D and E), the raw image was converted to a background-subtracted image, followed by applying the Threshold function using the Otsu algorithm to determine the optimal threshold based on the image histogram^22^. Then, Fill Holes function was used to accurately identify the area of cavitation or lesion in the region of interest (ROI). Next, the arbitrary shapes of the areas in the ROIs were selected by Analyze Particles function, and then the selected areas were calculated by Measure function. All areas were repeatedly measured three times. For the 2 MHz, cavitation dynamics were measured at multiple pulse numbers (1, 2, 3, 4, 5, 15, 30 and 50 pulses) to capture the detailed evolution of cavitation dynamics, whereas for the 3.5 MHz and 5 MHz conditions, cavitation dynamics were recorded only up to 5 pulses. PSH lesion area in the phantom was measured through the high-speed camera images after the PSH exposure. Additionally, cross-sectional view of the PSH lesion area in the phantom was observed using a microscopic imaging system (EVOS m7000, Thermo Fisher Scientific, MA, USA). Results were expressed as mean ± standard deviation (SD). During the experiments, a total of 15 tissue phantoms were used with five phantoms for each HIFU transducer (2 MHz, 3.5 MHz and 5 MHz). For a given PSH exposure condition, three repetitions were conducted. One-way ANOVA was performed to assess statistical differences among groups. In addition, linear regression analyses were conducted to evaluate the relationships between *t*_m_ and cavitation area or lesion area, as well as the correlation between cavitation area and lesion area under each condition.

## Electronic supplementary material

Below is the link to the electronic supplementary material.

Supplementary Material 1

Supplementary Material 2

Supplementary Material 3

Supplementary Material 4

Supplementary Material 5

Supplementary Material 6

Supplementary Material 7

## Data Availability

The data that support the findings of this study are available from the corresponding author upon reasonable request.
