# Supplementary figures and images for: A parametric study of bubble dynamics and lesion formation in liver tissue phantom during pressure-modulated shockwave histotripsy
Source: Sci Rep. 2025 Jul 21;15:26387. doi: 10.1038/s41598-025-11512-x (PMC12280012; doi:10.1038/s41598-025-11512-x)

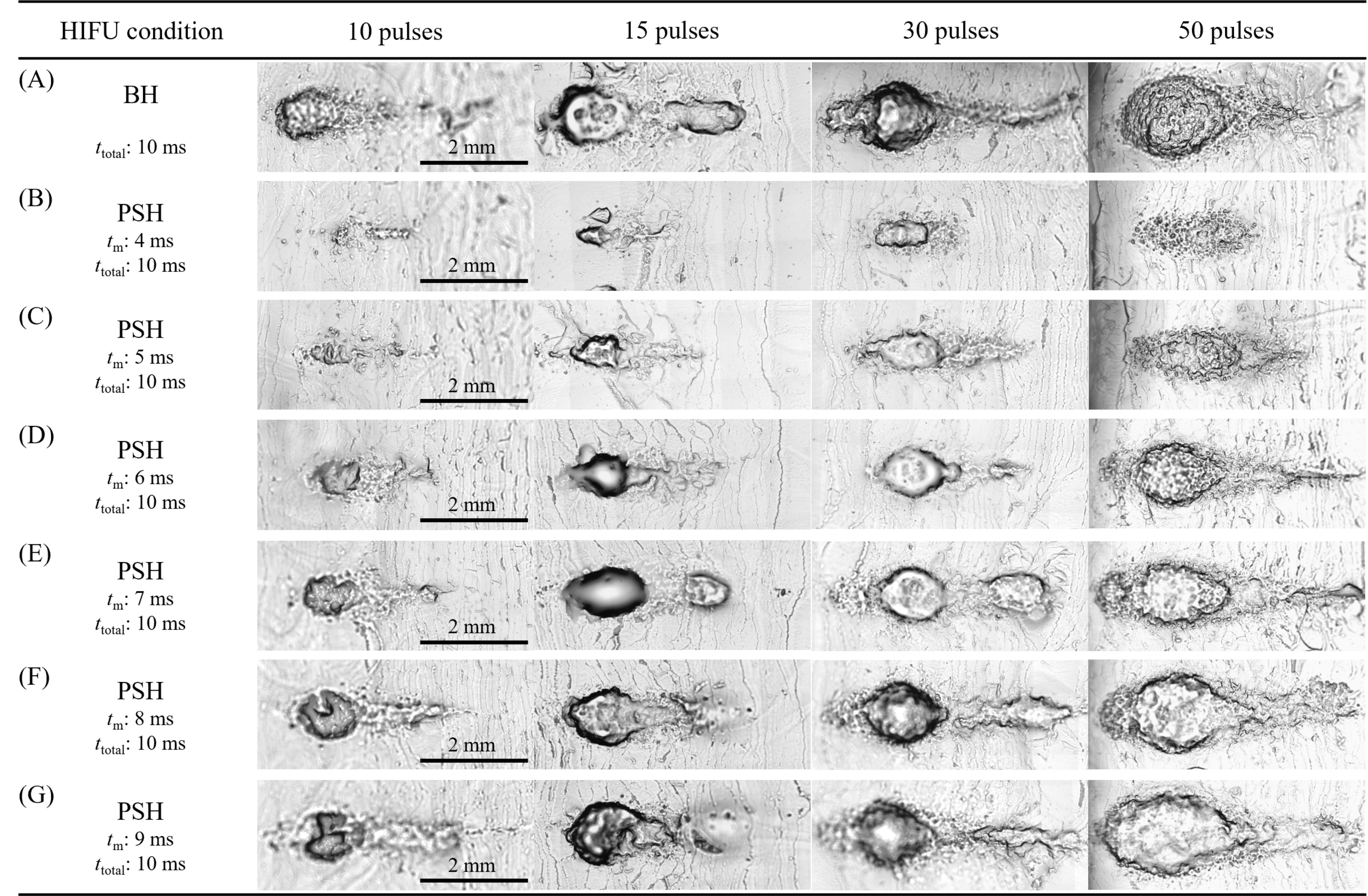

Supplement: Supplementary file 4 — Supplementary Material 4 [file 41598_2025_11512_MOESM4_ESM.tiff]

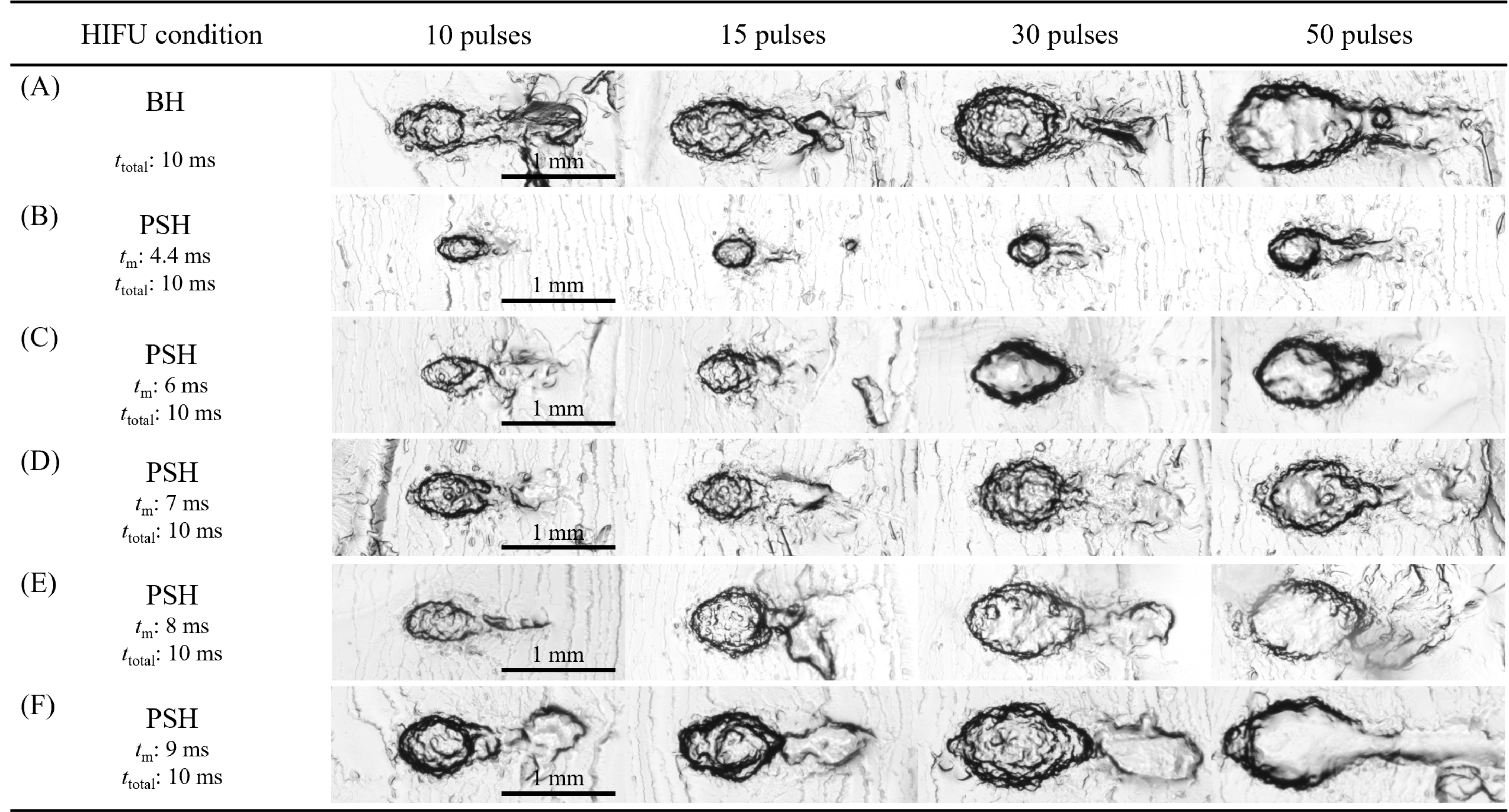

Supplement: Supplementary file 5 — Supplementary Material 5 [file 41598_2025_11512_MOESM5_ESM.tiff]

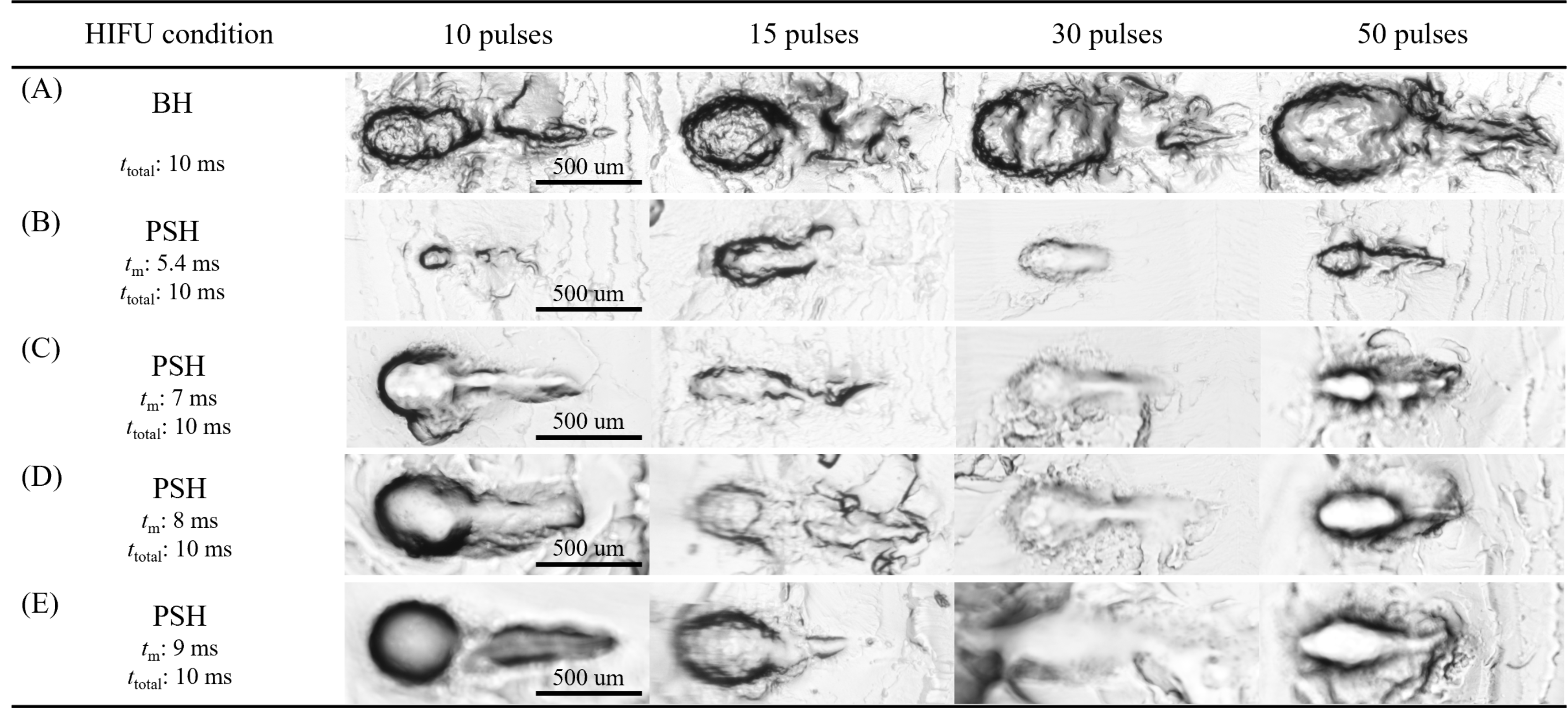

Supplement: Supplementary file 6 — Supplementary Material 6 [file 41598_2025_11512_MOESM6_ESM.tiff]
