# Supplementary material for: A parametric study of bubble dynamics and lesion formation in liver tissue phantom during pressure-modulated shockwave histotripsy
Source: Sci Rep. 2025 Jul 21;15:26387. doi: 10.1038/s41598-025-11512-x (PMC12280012; doi:10.1038/s41598-025-11512-x)
Supplement: Supplementary file 7 — Supplementary Material 7 [file 41598_2025_11512_MOESM7_ESM.docx]

Supplementary Materials

**A parametric study of bubble dynamics and lesion formation in tissue phantom during pressure-modulated shockwave histotripsy**

Jun Hong Park^a^, Jeongmin Heo^b^, Kisoo Pahk^c,*^, Ki Joo Pahk^d,*^

^a^Department of Radiology, Stanford University, Palo Alto, CA, 94304, USA.

^b^Bionics Research Center, Biomedical Research Institute, Korea Institute of Science and Technology (KIST), Seoul 02792, Republic of Korea.

^c^Department of Nuclear Medicine, Korea University College of Medicine, 73, Inchon-ro, Seongbuk-gu, Seoul, 02841, Republic of Korea

^d^Department of Biomedical Engineering, Kyung Hee University, Yongin, 17104, Republic of Korea.

*Corresponding author.

*Email address:* [kjpahk@khu.ac.kr](mailto:kjpahk@khu.ac.kr) (Ki Joo Pahk)

kisu99@korea.ac.kr (Kisoo Pahk)

**This PDF file includes:**

Supplementary Figs. S1 to S3 and Supplementary Video S1 to Video S3 legends


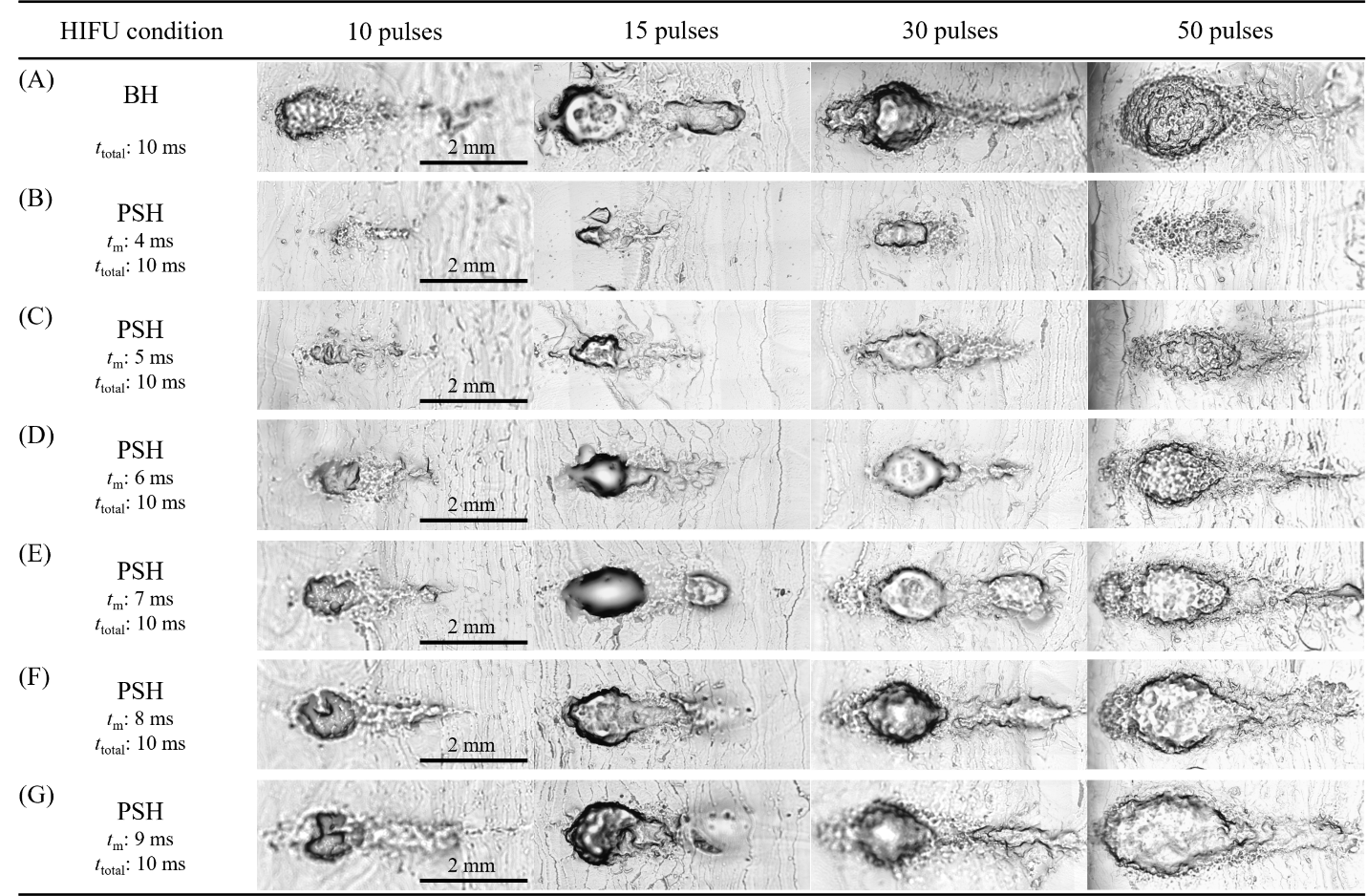


**Supplementary Figure S1.** BH and PSH lesions produced by the 2 MHz HIFU transducer with varying *t*_m_ and number of pulses. Cross-sectioned microscopic images of the lesion formation in the liver tissue phantom under various exposure conditions: (A) BH exposure conditions with a ttotal of 10 ms. PSH exposure conditions with tm of (B) 4 ms, (C) 5 ms, (D) 6 ms, (E) 7 ms, (F) 8 ms and (G) 9 ms with the number of pulses from 10, 15, 30 and 50 pulses. A scale bar indicates 2 mm.


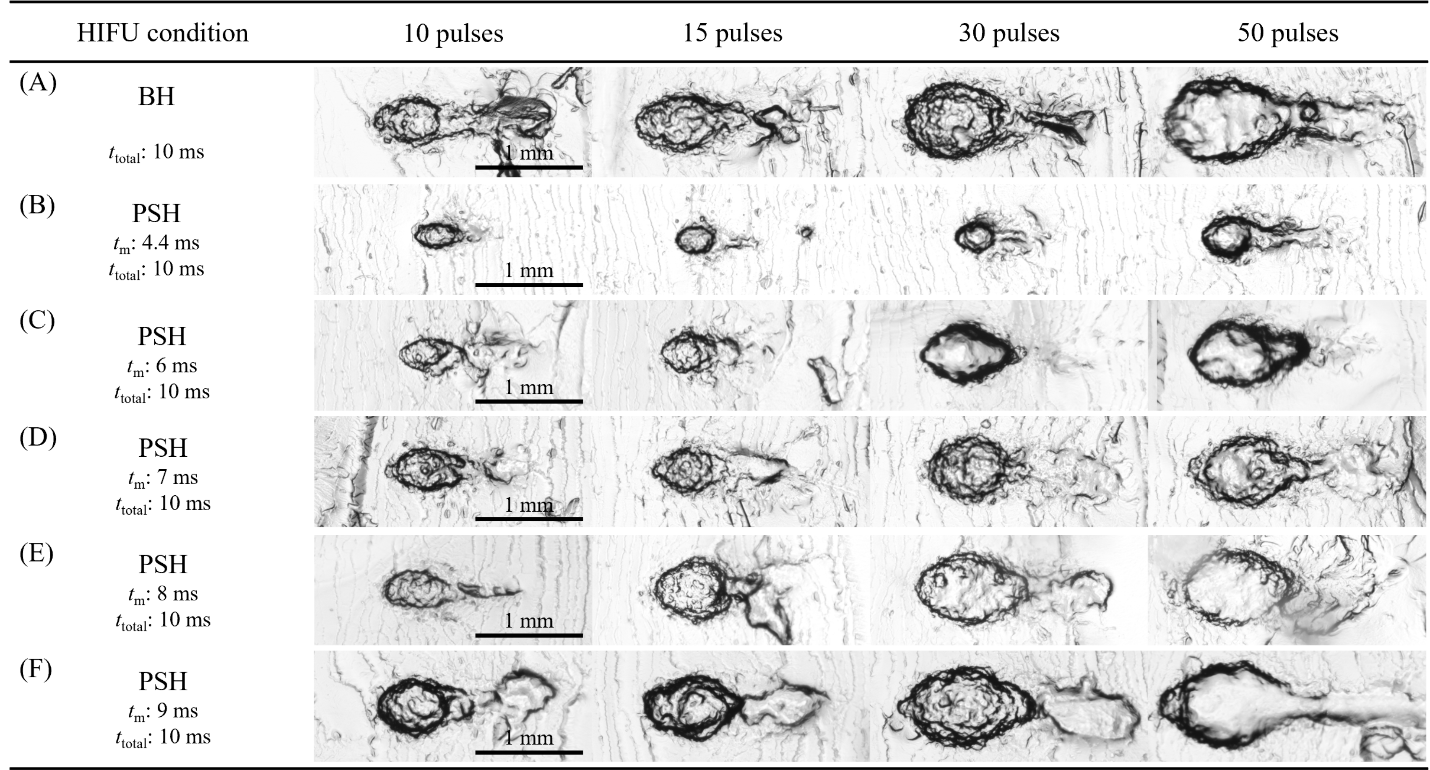


Supplementary Figure S2. BH and PSH lesions produced by the 3.5 MHz HIFU transducer with varying *t*_m_ and number of pulses. Cross-sectioned microscopic images of the lesion formation in the liver tissue phantom under various exposure conditions: (A) BH exposure conditions with a ttotal of 10 ms. PSH exposure conditions with tm of (B) 4.4 ms, (C) 6 ms, (D) 7 ms, (E) 8 ms and (F) 9 ms with the number of pulses from 10, 15, 30 and 50 pulses. A scale bar indicates 1 mm.


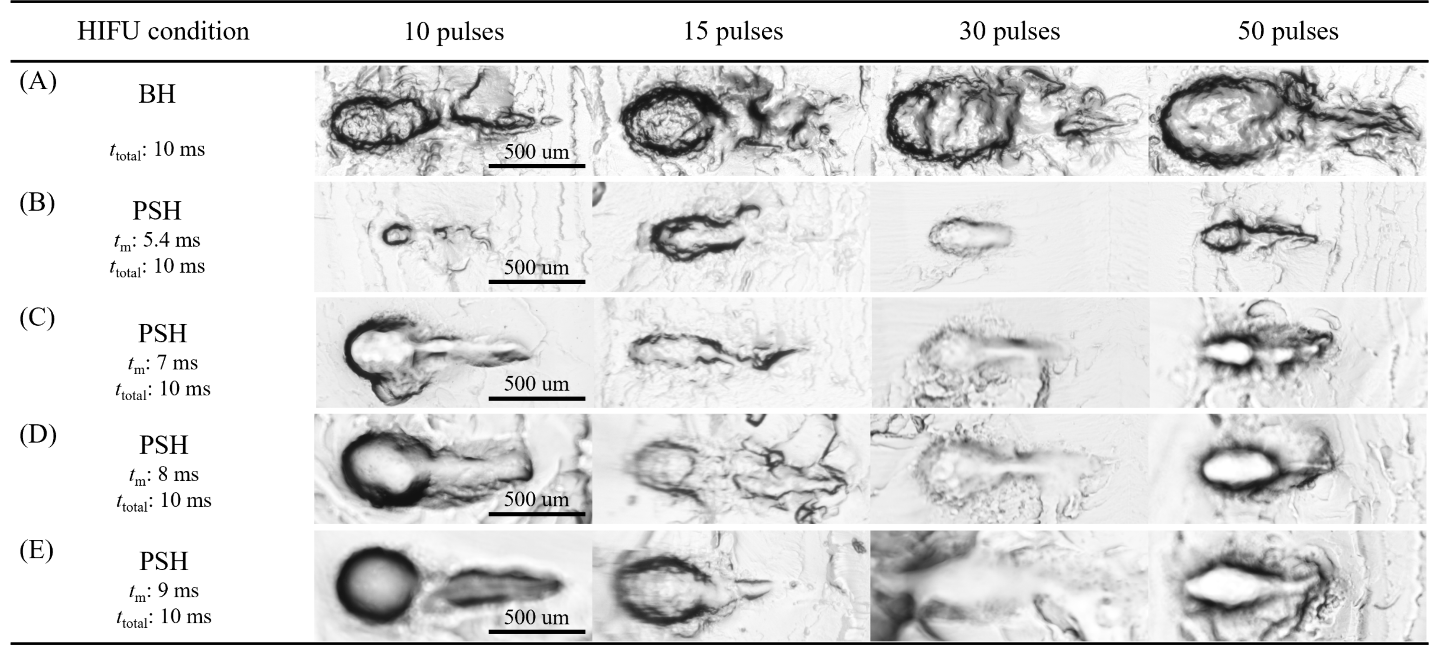


Supplementary Figure S3. BH and PSH lesions produced by the 5 MHz HIFU transducer with varying *t*_m_ and number of pulses. Cross-sectioned microscopic images of the lesion formation in the liver tissue phantom under various exposure conditions: (A) BH exposure conditions with a ttotal of 10 ms. PSH exposure conditions with tm of (B) 5.4 ms, (C) 7 ms, (D) 8 ms, and (E) 9 ms with the number of pulses from 10, 15, 30 and 50 pulses. A scale bar indicates 500 μm.

**Supplementary Video S1**. A movie showing the cavitation dynamics captured during the 2 MHz BH and PSH exposures

**Supplementary Video S2.** A movie showing the cavitation dynamics captured during the 3.5 MHz BH and PSH exposures.

**Supplementary Video S3**. A movie showing the cavitation dynamics captured during the 5 MHz BH and PSH exposures.
